# A Comprehensive Review of BET Protein Biochemistry, Physiology, and Pathological Roles

**DOI:** 10.3389/fphar.2022.818891

**Published:** 2022-03-25

**Authors:** Hafiz Akbar Ali, Yalan Li, Akram Hafiz Muhammad Bilal, Tingting Qin, Ziqiao Yuan, Wen Zhao

**Affiliations:** ^1^ Affiliated Cancer Hospital of Zhengzhou University, Zhengzhou University, Zhengzhou, China; ^2^ Key Laboratory of Advanced Drug Preparation Technologies, Ministry of Education, School of Pharmaceutical Sciences, Zhengzhou University, Zhengzhou, China; ^3^ State Key Laboratory of Esophageal Cancer Prevention and Treatment, Zhengzhou University, Zhengzhou, China

**Keywords:** BET proteins, epigenetics, Histone acetylation, BRD4, BET inhibitors

## Abstract

Epigenetic modifications, specifically acetylation of histone plays a decisive role in gene regulation and transcription of normal cellular mechanisms and pathological conditions. The bromodomain and extraterminal (BET) proteins (BRD2, BRD3, BRD4, and BRDT), being epigenetic readers, ligate to acetylated regions of histone and synchronize gene transcription. BET proteins are crucial for normal cellular processing as they control cell cycle progression, neurogenesis, differentiation, and maturation of erythroids and spermatogenesis, etc. Research-based evidence indicated that BET proteins (mainly BRD4) are associated with numeral pathological ailments, including cancer, inflammation, infections, renal diseases, and cardiac diseases. To counter the BET protein-related pathological conditions, there are some BET inhibitors developed and also under development. BET proteins are a topic of most research nowadays. This review, provides an ephemeral but comprehensive knowledge about BET proteins’ basic structure, biochemistry, physiological roles, and pathological conditions in which the role of BETs have been proven. This review also highlights the current and future approaches to pledge BET protein-related pathologies.

## 1 Introduction

Genes are found in compacted forms of chromosomes in histone. The covalent attachment of methyl, phosphoryl, acetyl, ubiquityl, and sumonyl groups to histone is called histone modification (HM) (Choudhary et al., 2009). These HMs lead to chromatin structure alterations, which ultimately change the gene expression. Inheritable chromatin variations in gene countenance, which are not dependent on DNA sequence are called epigenetic modification or epigenetics (Bird, 1986). Epigenetic modifications result in the formation of discrete chromatin states, (Allis and Jenuwein, 2016; Helai et al., 2019), 1) euchromatin in which chromosomes are loosely packed and 2) heterochromatin, where chromosomes are in the compact form (Bannister and Kouzarides, 2011). Epigenetic regulations include phosphorylation, acetylation, ubiquitination, and methylation. Three types of proteins have brought about the HMs changes. These are named writers, readers, and erasers. HMs play crucial and chain roles in signaling pathways (Dhalluin et al., 1999). In 1964, the HMs were discovered by Allfrey et al. (1964), and these modifications were lysine methylation and acetylation.

Bromodomains (BRDs) are groups of proteins described by John W. Tamkun et al. by observing the Drosophilia gene. They were named bromodomains as domain-containing gene Brahama (Tamkun et al., 1992). Bromodomains consist of 110 amino acids. BRDs attach loosely to lysine residues of acetylated proteins. The binding sites of BRDs at lysine are hydrophobic. Apart from N-terminal tails and the C-terminal domain, there are many other domains of BRDs that have distinct molecular functions. They are classified as methyltransferases, transcriptases, or transcription regulators, ATP-dependent chromatin remodelers, signal transducers, acetylases, and chromatin adaptors. They are responsible for protein metabolism and stability, cell cycle and signaling, chromatin remodeling and nuclear transport (Ray, 2010). The research studies over 2 decades confirm that BRDs are extensively studied and found to be irrationally regulated in various diseases, among which the most important is cancer. Others include cardiovascular disease (CVD), neurodegenerative disease and inflammatory diseases (Pérez-Salvia and Esteller, 2017; Zaware and Zhou, 2017).

## 2 Bromodomains and extra terminal domain bromodomains

### 2.1 Structure

In the human genome, there are 61 BRD proteins divided into eight structural groups (based on x-ray crystal) and 46 families of proteins. All have common four alpha-helical bundles named as αZ, αA, αB, and αC, and two interhelical loops recognized by the ZA loop and BC loop. The BC loop link αA and αC, while the ZA loop binds αB and αZ helix. There is very little similarity in sequence in N- and C-terminal regions outside the main two helices. The HMs occur when histone is acetylated at the eight N-terminal tails of the core. Acetylation and other HMs, such as ubiquitination and methylation, yield a compact set of histone codes that control the expression and regulation of genes. HMs are responsible for many important metabolic processes. Among the HMs, the most studied epigenetic readers are BRDs. There are 61 BRDs in the human genome, which are distributed among 46 types of different proteins. The acetyl-lysine (KAc) is hydrophobic, and asparagine is present at 48 BRDs, while the remaining 13 BRDs have aspartate, tyrosine, or threonine. The asparagine bearing BRDs are classified as typical BRDs, while the others are called atypical BRDs (Okada et al., 2005; Jeffers et al., 2017). BET proteins belong to the fifth group of the BRD family. BETs include BRD2, BRD3, BRD4, and BRDT. BETs have two tandems, i.e., BD1 and BD2 and an extraterminal domain. BETs perform protein transcription, transcription elongation, and chromatin opening. BETs bind to the di-acetylated lysine of proteins. This distinguishes BETs from other BRDs. There is a slight difference of four amino acids in BD1 and BD2. The BD1 domain consists of Gln85, Asp144, Lys141, and lle146, and the BD2 domain consists of Lys374, Val435, Pro430, and His433 instead (Mujtaba et al., 2007; Nicodeme et al., 2010; Lloyd and Glass, 2018). The tyrosine (Tyr) and asparagine (Asn) amino acid sequences of all BRDs are preserved (Pérez-Salvia and Esteller, 2017; Zaware and Zhou, 2017).

The BRDs are classified into nine major families depending upon the functions in cells (Zeng and Zhou, 2002; Filippakopoulos et al., 2012). These are:.

I. (IA) Histone acetyltransferases (HATs)-containing transcriptional cofactors (PCAF, p300/CBP). HAT-containing transcriptional cofactors are responsible for erythroid gene regulation development, initiation, activation, and assembly of regulatory enhancers or mediator complex at target genes (Ortega et al., 2018).
(IB) BRPF1/2/3 (bromodomain and PHD finger-containing protein) and BRD8 BRPF1is are responsible for HOX gene expression by augmented acetylation of MOZ *via* BRD (Ullah et al., 2008). BRD8 mediates nuclear receptor translation when it binds to beta receptors of the thyroid or alpha receptors of retinoid X (Monden et al., 1999).
II. SET domain holding MLL and histone lysine methyltransferases (HKMTs) are assembled in it. The major function of this group of proteins is chromatin remodeling.
III. (IIIA) Chromatin remodeling factors (BRD9, SMARCA2/4, and PBRM1) are key components of mammalian ATP-dependent chromatin remodeling complexes (Mashtalir et al., 2018).
(IIIB) ISWI family chromatin remodeling complex possesses nucleosome assembly and spacing, DNA helicase activity, and heterochromatin and chromosome segregation activity in DNA replication (Xiao et al., 2009).
IV. AAA domain-containing ATPase family proteins, such as ATAD2. These are engaged in the reassembly of chromatin and DNA replication. They ligate to newly synthesized di acetylated histone (Koo et al., 2016).
V. BET family proteins (BRD2, BRD3, BRD4, and BRDT) are grouped in the fifth class. They are transcription coactivators, facilitate chromatin opening, recruit transcription factors and coactivators to target gene promoters and enhancers, and activate paused RNA polymerase II complexes to promote transcription elongation (Zhang et al., 2016)
VI. Group six BRD proteins are called TIF1 (transcriptional intermediary factor 1). TIF1 alpha is responsible for p53 stabilization, and TIF1 beta promotes heterochromatin formation (Jain and Barton, 2009) (Zeng et al., 2008). TIF1 gamma possesses E3 SUMO/ubiquitin ligases properties, assists in remodeling of the nucleosome, stops the proliferation of tumor cells and tumorigenesis, and helps in degradation of tumor (Xue et al., 2015).
VII. This group contains sparkled protein (SP) family, such as SP100, SP110 etc. These proteins are involved in stress stimuli reaction to cells as they are part of subnuclear bodies of promyelocytic leukemia (PML-NB) (Seeler et al., 2001). These proteins interact with DNA and other proteins and maintain chromatin structure (Bottomley et al., 2001).
VIII. DEAF-1 proteins, such as ZMYDN8/11 are categorized in this group. These proteins act as transcriptional corepressors, regulators, control gene expression, and protect DNA from damage (Savitsky et al., 2016).
IX. WD-repeat proteins, such as BRWD1 (WDR9), PHP (WDR11), and BRWD3, are assembled in this group. They perform many cellular functions, such as cell cycle regulation, RNA processing, cytoskeleton assembly, transcription, and signal transduction (Li and Roberts, 2001).

The most studied and discussed family of BRDs is bromodomains and extraterminal domains (BETs). This family of BRDs is branded to contain an extraterminal (ET) domain and two tandem bromodomains. BET proteins are associated with mitotic chromosomes throughout the cell cycle (Reyes-Garau et al., 2019). There are four members of this family, namely, BRD2, BRD3, BRD4, and BRDt. This family has a common architectural domain possessing an ET and two N-terminal bromodomains. BRD4 and BRDt have extra C-terminal domains (CTD). The N-terminal domains (BD1, BD2) are the sites that interact with acetylated lysine through a hydrophobic region (Dhalluin et al., 1999). The BDs are hydrophobic regions of amino acids, which are involved in protein–protein interactions and act as “readers” of epigenetics. Transcriptional recruitments are brought out by ET, and CTD is involved in the recruitment of positive elongation factor (P-TEFb). BET proteins ligate to histone at acetylated lysine regions located in “super-enhancers” (DNA region enriched with repressive acetylated H3K27 marks and RNA polymerase II, or promoter regions of the genes) (Barrero, 2017; White et al., 2019). All the BET members recruit transcriptional cofactors through the ET domain and facilitate transcription (Shi et al., 2013). BET proteins are sturdily concerned with regulation, growth, differentiation of cells, and inflammation. Chromosomal location of BET family BRDs in the human genome is near to the Notch genes. The BRD2 place is on chromosome 6 along with the Notch 4 gene, BRD3 resides on the 9th chromosome beside Notch1, BRD4 exists at the 19th chromosome with the Notch 3 gene, and BRDT is found on the 1st chromosome along with the Notch 2 gene. The coexistence of BET and Notch genes at the human genome indicates functional resemblance among these genes (Houzelstein et al., 2002; Wu and Chiang, 2007).

BET proteins dislocate the HEXIM/7SK snRNP (factor 1) transcription factor from cyclin T1/CDK9 (factor 2) of P-TEFb, which permits the RNA Pol II activation through serine 2 phosphorylation (Wu et al., 2013; Winter et al., 2017). BRD2, BRD3, and BRD4 regulate transcription at histone tails, while BRDt is expressed in germ cells. They primarily exist in the nucleus but are functionally not confined to cells. Besides being directly involved in transcription, the BET member also stimulates transcription by nucleosome hyperacetylation, thus, acting as a “histone chaperone” (LeRoy et al., 2008; Kanno et al., 2014). Apart from histone, they can recognize other acetylated proteins and transcription factors, and involve in their transcription, e.g., mesodermal-forming transcription factor TWIST (Shi et al., 2014).

## 3 Cellular functions of bromodomain and extraterminal protein family

### 3.1 BRD2

BRD2, previously known as RING3 (really interesting new gene 3) or FSHRG1 (female sterile homeotic related gene 1), stimulates the activity of E2F1 and E2F2 proteins, which promote the synthesis of proteins needed for the G1/S phase. BRD2 is the first nuclear protein, which translates through G-protein-coupled receptors (called canonical protein) (Denis et al., 2000). BRD2 is expressed in the mammary glands, ovaries and testes, uterus, epididymis, and a number of other tissues. The study of Gyuris et al. discovered the vital role of BRD2 in neural tube closure and dorsal root completion during embryogenesis. BRD2 manifestation is essential for neurogenesis and maximum during neuronal tube growth and closure. Pleiotrophin (Ptn) is a neuronal growth factor responsible for neuroprotection, nerve regeneration, and growth of the nervous system. It is highly expressed during nervous system development. During the process of neuronal differentiation, Ptn interrelates with BRD2 and improves the cell-differentiating and stimulating activity of BRD2 (Padmanabhan et al., 2016).

Data obtained from fluorescence resonance energy transfer (FRET) technology revealed that BRD2 has a strong affinity toward acetylated K12 of histone H4 (Padmanabhan et al., 2016), promotes gene transcription, elongation, and chromatin binding in coordination with BRD3. Nuclear serine and theorine (Ser/Thr) activities are enhanced during cellular proliferation. BRD2 possesses nuclear Ser/Thr kinase properties (Denis and Green, 1996). In humans, BRD2 and BRD4 remain persistent with chromatin throughout the cell cycle, thus, involving in epigenetic memory (Kanno et al., 2004). It attaches to the H3 and H4 regions of acetylated histone and activates transcription by recruiting transcriptional coactivators, factors, and transcriptional repressors. Five transcription complexes possess BRD2 and a factory of chromatin remodeling (Denis et al., 2006). These are 1) TATA binding factor-associated factors and Pol II, 2) activated transcription factors E2F and DP-1, 3) mediator proteins, 4) chromatin/histone modification enzymes (HDAC11, CBP, and p300), and 5) SWI/SNF remodeling complex components These transcription factors in association with BRD3 brought about Pol II transcription at the acetylated nucleosome. E2F1 and E2F2 are major cell cycle S phase gene transcription regulators, and BRD2 recruits these factors (Lovén et al., 2013). BRD2 controls the expression of transcriptional regulators, cyclin E, D1, and A. Cyclin D1 and E expressions are critical for mitotic cells, and cyclin A for B lymphocyte-proliferating cells. BRD2 controls the expression of GATA1 (erythroid transcription factor) and functions in the maturation of the erythroid (Stonestrom et al., 2015). Aron et al. reported that BRD2 is a dire component of chromatin and plays an important role in the neurogenesis and embryogenesis of mammals (Gyuris et al., 2009). A study by Ruxin et al. established the fact that BRD2 improves insulin signaling and metabolic disorders (Sun et al., 2017). Among BETs, BRD2 is the main BRD that is involved in the activation and regulation of NF-κB-mediated inflammatory response (Gallagher et al., 2014).

### 3.2 BRD3

BRD3, also called ORFX or FSHRG2 gene, interacts with acetylated lysine residues of an erythroid transcription factor or GATA1 factor of transcription. GATA1 controls the expression of erythroid and megakaryocytes-specific genes (Lamonica et al., 2011). Cyclin D1 is vital for the transition of dividing cells from the G1 to S phase. BRD3 is a transcriptional regulator of cyclin D1. BRD3 and BRD2 possess properties of nucleosome assembly followed by DNA replication, a process called “nucleosome chaperone.” This nucleosome chaperone initiation by BRD2 and BRD3 *via* hyperacetylated nucleosome causes elongation of RNA Pol II (Gamsjaeger et al., 2011).

### 3.3 BRDT

BRDt, a testis-specific bromodomain protein is expressed specifically in the testes (Shang et al., 2004). During meiosis of spermatogenesis in the male germline, BRDt expression starts which lasts until the postmeiotic stage (Gaucher et al., 2012). Shang et al. used BRDt homozygous male mice for testicular histology and found that deletion of BRDt protein results in sterility, oligospermia, and abnormal sperm morphology (Shang et al., 2007). Cyclin A1 gene is a regulatory gene in male germ cell lines and is necessary for spermatocytes to enter the first meiotic division. Cyclin A1 expression is initiated and controlled by BRDt (Liu et al., 1998; Nickerson et al., 2007). H1t is a testis-specific histone, which expression is repressed by BRDt during spermatogenesis. BRDt represses the H1t by interacting with tripartite motif-containing 28 proteins (TRIM 28), histone deacetylase (HDAC1), PRMT5, and arginine-specific histone methyltransferase 5 (Wang and Wolgemuth, 2016). BRDt, in association with other regulatory factor proteins, performs a transcriptional repressor role or activator role during spermatogenesis. The C-terminal motif sequences of BRDt and BRD4 have similarities to each other. Cdk 9 and cyclin T1 are heterodimers of positive transcription elongation factor (P-TEFb), which binds the C-terminal motif of BRD4 (Morinière et al., 2009). BRDt is necessary for the recruitment of P-TEFb with BRD4. BRDt is responsible for chromatin remodeling by interacting with hyperacetylated nucleosomes (Bisgrove et al., 2007).

### 3.4 BRD4

BRD4, originally known as mitotic chromosome-associated protein (MCAP) or FSGRG4 or Hunk1 (Dey et al., 2000). BRD4 is present almost in all tissues, mainly confined to the cell nucleus. It has 80% amino acid identical similarity with BRD2. BRD4 plays a pivotal role in the control of cell cycle, embryogenesis, and stabilizing the genome (Wu and Chiang, 2007). BRD4 recruits P-TEFb, a transcription factor, for phosphorylation of RNA polymerase II at transcriptional start sites and persuade transcription elongation. BRD4 recruits P-TEFb complexes, which consequently phosphorylate RNA Pol II and NELF (negative elongation factor) leading to the start of transcription elongation (Jang et al., 2005; Bisgrove et al., 2007). BRD4 and mediator complex reside in the same enhancer subset, which is called “super-enhancers (SEs).” SEs are rich in acetylated lysine 27 histone 3 (H3), which favors the BRD4–MED functional interactions. SEs are crucial for lineage-specific and growth survival-promoting genes transcription (Lovén et al., 2013; Filippakopoulos and Knapp, 2014; Bhagwat et al., 2016). The role of BRD4 as a transcriptional regulator is obvious as it interacts with 1) CDK9 and cyclin T1, which are active forms of P-TEFb, and 2) 30 subunits of mediator complex coactivators that interrelates BRD4 and P-TEFb physically. Both the mediator complex and BRD4 support each other and help to recruit P-TEFb at the genome. BRD4 interrelates P-TEFb in two distinct ways (Jang et al., 2005): 1) BD2 module of BRD4 specifically recognizes and interacts with cyclin T1 at the promotor region of active genes, which maintains the Pol II, and leads to initiation and elongation of transcription in cell growth and development (Mochizuki et al., 2008; Yang et al., 2008), 2) CTM of BRD4 binds to T1 and CDK9. CDK9 kinase activity facilitates RNA Pol II transcription elongation *via* phosphorylation, while inhibiting negative regulators of RNA Pol II (Zhou et al., 2012). BRD4 remains attached to the chromosome throughout mitosis when most of the nuclear factors are scattered in the cytoplasm. This is apparent from the presence of BRD4 in euchromatin but not present at the centromere. Cyclin, CDK9, and other mediating factors are activated by BRD4 in the G2/M phase, which indicates that BRD4 plays a role in controlling cell cycle regulation as the cell must enter into mitosis (Dey et al., 2000). Extraterminal domain (ETD) or extraterminal motif (ETM) of BRD4 enrolls transcription activators, such as acetyltransferase (e.g., P300), histone arginine demethylase (e.g., JMJD6), and histone methyltransferase (e.g., NSD3). BRD4 has unique properties as it can ligate to other acetylated proteins also, such as transcription factors (Alpatov et al., 2014). BRD4 possesses kinase activity. ETD can attach to SWI-SNF and CHD2, which are responsible for ATP-dependent chromatin remodeling (Rahman et al., 2011; Shi et al., 2013).

#### 3.4.1 Role of BRD4 in cell cycle progression

Signal-induced proliferation-associated gene-1 or SPA-1 is mainly expressed in lymphocytes in fetal and adult lymphohematopoietic tissues. The G2 phase is somehow controlled by SPA-1 gene expression in coordination with BRD4. BRD4 is critical from G2 to M transition during the cell cycle. Knockout of BRD4 from cells leads to G2/M phase arrest of the cell cycle. This is due to the lack of balance between SPA-1 and BRD4 activity, which is necessary for cell division (Dey et al., 2000). BRD4 is crucial for the cell cycle as it plays a pivot role for entry from the G1 phase to the S phase during mitosis. A study by Kazuki et al. demonstrated that knockdown of BRD4 from dividing cells resulted in the inactivation of many G1 genes and arrested the cells at the G1 phase, whereas in control cells, the G1 genes were well expressed, and the cells enter the S phase and progressed well (Mochizuki et al., 2008).

BRD4 initiates phosphorylation of histone at H3 in early chromosomal condensation. Its expression is high during the Go/G1 phase in the cell cycle. BRD4 remained confined to the nucleus in the M phase even when most of the nuclear factors are released in the cytoplasm during transcription. BRD4 recruits P-TEFb-dependent serine 2 phosphorylation at the C-terminal at telophase and serves as a transcriptional cofactor. This signifies Pol II elongation status. BRD4 and RNA Pol II recruiting at interphase accelerates the synthesis of mRNA and facilitates transcription reactivation in postmitotic cells. M/G1 genes are expressed during cellular division and play an important role in cell–cell interaction (Zhao et al., 2011). M/G1 genes are programmed to express at the mitosis end or after mitosis immediately. BRD4 is kept attached to m/G1 gene transcription sites and marks the M/G1 genes for transcription memory in mitosis, which then initiate M/G1 transcription in late mitotic postmitotic cells and daughter cells (Dey et al., 2009).

IEGs are immediate–early genes activated rapidly in external stimuli response for the consolidation of synaptic modifications and synaptic memory formation. BRD4 is critical for neuronal development and arbitrates transcriptional regulation essential for learning behavior and memory. Under the heat stress stimuli, heat shock retort is introduced, which is accomplished by the production of heat shock proteins and partial inhibition of intron removal (RNA splicing). BRD4 preserves intron and averts cells from heat tempted splicing inhibition. Thus, BRD4 controls gene expression under external stimuli (Patel et al., 2013; Hussong et al., 2017).

#### 3.4.2 BRD4 is present in distinct forms of positive transcription elongation factor b complexes

P-TEFb, a heterodimer of cyclin-dependent kinase 9 (CDK9), is present in three diverse complex forms (Yang et al., 2005). BRD4, CDK9, and cyclinT1 form the core component of P-TEFb (Yang et al., 2005). CDK9, AFF1/AFF4, cyclin T1, ELL1/ELL2, and ENL/AF9 make the other P-TEFb complex called “super elongation complex” (SEC) (Liang et al., 2018). CDK9, cyclin T1, MEPCE, HEXIM1/2, LARP7, and 7SK RNP (ribonucleoprotein complex) together make the final and third P-TEFb complex (Fujinaga, 2020). BRD4 performs various parts in stimulating Pol II-dependent transcription at chromatin and DNA levels. Cyclin/T-Cdk9, an activated P-TEFb complex, accounts for about half the quantity of P-TEFb in cells. The other half of P-TEFb is a repressive complex, called cyclin T-Cdk9-HEXIM1-7SK. BRD4 binds to the active P-TEFb form rather than the repressor complex. This indicates strongly that BRD4 is involved in transcription (Wada et al., 1998; Yamada et al., 2006).

#### 3.4.3 BRD4 is found in a human papillomavirus transcriptional silencing complex

E2 is a human papillomavirus (HPV) gene that controls viral DNA replication, transcription, segregation, and genome maintenance (Wu et al., 2006). In humans, E2 represses the viral gene expression, along with E6 and E7 (which are oncoproteins) expression, which antagonizes the pRB and p53 tumor suppressor genes. BRD4 recruits E2 at acetylated histone and functions as a transcriptional regulator in viruses. BRD4 links HPV genome segregation in the viral mitotic division (You et al., 2004; Abbate et al., 2006).

#### 3.4.4 BRD4 as transcriptional initiator

P-TEFb, through its complexes CDK9 and cyclin T1, allows the RNA Pol II entry to the transcription site and prevents the pause of gene expression. The active form of P-TEFb phosphorylates NELF (negative elongation factor complex, a pausing factor), binds with cofactors and TFs, and hinders their binding capability to chromatin leading to C-terminal domain of RNA Pol II phosphorylation at transcriptional activation. BRD4 not only recruits P-TEFb but also phosphorylates its active motif CDK9 (Kanno et al., 2014). BRD4 arbitrates the 7SK snRNP/HEXIM release, which facilitates the generation of transcription initiation complex and RNA Pol II pause release (Jang et al., 2005; Devaiah et al., 2012). BRD4 averts the interaction of P-TEFb with 7SK/HEXIM (an inhibitory ribonucleoprotein) preventing P-TEFb from its inactive form. BRD4 acts as a stopping site for P-TEFb at active hyperacetylated transcriptional start sites at (TSSs) leading to the release and activation of Pol II into elongation. BRD4 also facilitates transcription elongation without recruitment of P-TEFb (Rahman et al., 2011). BRD4 phosphorylates RNA Pol II resulting in topoisomerase I activation, which facilitates RNA Pol II transition progression and DNA decompaction; thus, BRD4 presents kinase activity (Devaiah et al., 2012). Transcription mediators are a group of protein complexes that transmit signals from activators and transcription factors (TFs) to the promoters. One of the key mediators is PIC (preinitiation complex); enhancers influence the assembly of PIC and control the PIC assembly of regulatory proteins and TFs. Transcription initiates by the recruitment of RNA Pol II at PIC on the promoter region of the genome followed by phosphorylation of RNA Pol II at serine 2. BRD4 being a cofactor of mediator complex is present at active enhancers and super-enhancers (Dey et al., 2000). BRD4 interacts with histone modifiers and TFs by both ET domains and BDs. BRD4’s ET domain interacts with CDH4 and SWIF/SNF remodeling nucleosome enzymes, JMJD6 (Jumanji C-terminal-containing protein arginine demethylase) and NSD3 lysine methyltransferase, such as histone modifiers, and help in transcription regulation (Angrand et al., 2001). These interactions of BRD4 accelerate the synthesis of mRNA and facilitate the decompaction of chromatin so that transcription is activated (Liu et al., 2013; Yang et al., 2020). BET proteins being TFs directly participate in the differentiation of inflammatory CD4 T lymphocytes to T1 helper cells, such as Th1, Th2, and Th17 cells. BRD4 specifically involves the differentiation of naive CD4 lymphocytes into Th17 cells (Winter et al., 2017). The histone acetyltransferase (HAT) activity pattern of BRD4 is different from classic HATs as it acetylase H3 and H4 K122 leading to the expulsion of nucleosome and decompaction of chromatin, which results in the acceleration of transcription (Kanno et al., 2004).

#### 3.4.5 BRD4 and transcription regulation

BRD4 regulates transcription by transcription factor (TF) recruitment. Studies by Wu et al. showed that BRD4 can also interact with non-acetylated C-Jun, AP2, Myc, YYA, V/EBPbeta, and p53, such as TFs (Wu et al., 2016). BRD4 interacts with transcription genes that are necessary for transcription in the G1 phase of mitosis, hence, ensuring cell cycle progression. Embryonic stem cells (ESCs) and pluripotency in early embryogenesis are maintained by BRD4 through “Nanog, OCT4, and PRDM1,” such as ESC transcription factors at super-enhancer regions (Di Micco et al., 2014). BRD4 depletion reduces the ESC differentiation capacity to multiple cells, induces neuroectodermal linage, and EMT (epithelial-to-mesenchymal transition) (Di Micco et al., 2014). BRD4 expression is necessary for myogenesis and adipose tissue development. A study by Najafowa et al. demonstrates that the whole osteoblast differentiation procedure from mineralization to bone formation is under BRD4 supervision. Casein kinase II (CKII) activates neuronal stimulation and involves in memory formation. BRD4 regulates CKII-mediated transcription, which helps in learning behavior and memory creation (Korb et al., 2015).

#### 3.4.6 BRD4 as DNA damage repairer

BRD4 is not only one of the leader regulators of the DNA repair system but also a direct contributor of DNA double-strand break (DSB) repair in the conventional and uncanonical way. A study of BRD4 and prostate cancer states that ionizing radiation (IR) induces DSBs, which is repaired by BRD4. This study also indicated that DSBs repair activity of BRD4 is independent of its transcriptional activity but based on its ability to bind with DNA repair motifs and histone modification (Korb et al., 2015). Hyperacetylation of Histone H4 and phosphorylation of H2AX (γH2AX) are hallmarks of DSBs. These amendments at each end of the RNA strand induce recruitment of BRD4, which act as cutting-edge sites for DNA repair complex. P53 binging protein (53BPI) being a vital DNA repair part, provides a molecular framework and recruits the additional DSB repair response protein at the damaged site. 53BPI is a binding partner of BRD4 (Stanlie et al., 2014). BRD4 is also involved in DNA damage checkpoints. CDC6 factor is an activator of replication checkpoint response. BRD4 interrelates the CDC6 factor and regulates its function in DNA replication stress. Replication stress activates checkpoint kinase 1 (CHK1), which is a stress DNA replication marker and checkpoint for DNA damage. Activation of CHK1 leads to malfunctioning of other DAN damage checkpoint factors, which results in DNA DSBs and chromosomal rearrangement (Zhang et al., 2018). Telomere shortening occurs at each mitosis and sense aging when telomer becomes very thin. Telomerase reverse transcriptase controls the activity of the telomer. Abnormal lengthening of telomer due to alteration in expression of telomerase is a hallmark of cancerous cells. On one side, though, inactivity of telomerase expression leads to “replicative senescence” (permanent cell growth arrest) due to telomere erosion in growing mitotic cells. Telomerase reverse transcriptase expresses in a controlled and timely manner and hinders too shortening of telomers. BRD4 has telomere-maintaining properties as it recruits the telomerase and telomerase-associated complexes at short telomer, facilitating lengthening (in a controlled manner) and stabilizing of the telomer by accumulating at the chromosomes’ end of histone acetylation (Zhao et al., 2000; Zhang et al., 2003).

## 4 Knockdown or inhibitory effects of bromodomain and extraterminal proteins

BRD2 and BRD4 regulate the body’s energy-providing genes, such as the β cells of the pancreas, and knockdown or decrease the expression of BRD2 improving β-cell function by increasing insulin secretion and protects the cells from insulin resistance (Wang et al., 2013; Deeney et al., 2016). Depletion of BRD2 at the promoter region increases adiposity and embryonic mortality (Wang et al., 2009). BRD4 diminution prejudices the immune system lineage development (Houzelstein et al., 2002). Libor et al. experimentation revealed BRD2’s involvement in developing seizures by altering the GABA system, which leads to IGE (idiopathic generalized epilepsy) (Velíšek et al., 2011). BRD2, BRD3, and BRD4 play roles in erythroid development, maturation, and differentiation independently and in a coregulatory manner. BRD2 and BRD4 depletion inhibit erythroid maturation; BRD3 depletion has minimal effect on erythroid maturation, but both BRD2 and BRD3 depletion results in the complete inability of erythroid maturation (Stonestrom et al., 2016). BRD2 activates and regulates NF-κB, which is a key factor of inflammatory cascades leading to cancer progression (Gallagher et al., 2014). Knockdown of BRD2 from embryo results in the inadequate expression of genes essential for neuronal development, failing to enter a midgestation period, head and tail region imperfection, and neuronal tube flaws, which ultimately lead to death within 15 days (Padmanabhan et al., 2016). Knockdown studies of BRD2 in mice have demonstrated the increased expression of inflammatory mediators and induction of obesity characterized by hepatosteatosis and hyperinsulinemia, but the whole-body metabolic profile is upgraded like blood glucose level lowering, increased glucose tolerance, and increased brown fat mass. The improvement in whole-body metabolic contour assisted the body to evade type 2 diabetes (Belkina et al., 2013; Wang et al., 2013).

## 5 Pathological roles of bromodomain and extraterminal proteins

Several studies demonstrate the role of the BET bromodomain family in disease development and progression. BET proteins are dysregulated in inflammation, cancer, metabolic disorders, neurodegenerative disorders, renal diseases, lung diseases, etc.; the most studied protein in a pathological sense is BRD4 (Grivennikov et al., 2010). BET proteins promote the expression of oncogenes by the possession of aberrant chromatin structure in various types of cancers, such as AML (acute myelogenous leukemia), BL (Burkitt lymphoma), MM (multiple myeloma), etc. (Zuber et al., 2011; Ott et al., 2012; Zhang et al., 2020). BRD2 overexpression in cooperation with BRD4 causes chromatin decompaction and androgen receptor activation, which lead to prostate cancer development (Devaiah et al., 2016). Deregulation of BET proteins not only initiates solid and hematopoietic malignancies but also maintains and helps to progress their cancerous phenotypic activities (Kanno et al., 2014). Idiopathic generalized epilepsy (IGE) occurs among 30% of total epilepsies. BRD2 is involved in the development of IGE through spontaneous seizure advancement, anatomical γ-aminobutyric acid (GABA) system deterioration, and sex-related seizure susceptibility surge (Velíšek et al., 2011).

Cells produce cytoprotective substances, upregulate cytoprotective genes, and inhibit splicing to cope with heat stress stimuli. BRD4 is involved in the splicing process. BRD4 binds with HSF1 (heat shock factor 1) in heat stress, BRD4 is engaged in SatIII (noncoding), and RNA transcription is upregulated, which increases the splicing process (Hussong et al., 2017).

### 5.1 Bromodomain and extraterminal proteins in cancer

Initiation of tumor formation is the foremost footstep in cancer development. Tumors contain a population of neoplastic and non-neoplastic cells that produce tumor microenvironment (TME). TMEs consist of immune cells, inflammatory cells, stromal cells, endothelial cells, fibroblasts, bone marrow-derived inflammatory cells, etc. These cells promote consistent signaling, migration, invasion, and blood vessel growth in tumor and adjacent cells. BRD4, as an epigenetic reader, plays a straight role in tumorigenesis by gene expression regulation of neoplastic and non-neoplastic cells (McConkey et al., 2010; Hussong et al., 2017). MYC or c-MYC is a proto-oncogene that regulates numeral cellular functions including cell cycle, a transformation of cells and apoptosis, etc. It attaches to promoter sites of acetylated histone lysine and *via* recruitment of P-TEFb and other mediator factors and potentiate or overexpress the genes resulting in amplified transcription, activation, and elongation (Frank et al., 2003; Lin et al., 2012). MYC expression is augmented in 60%–70% of cancers, and it is mainly regulated by BET proteins (Dang, 2012). Anomalous expression of c-MYC provides cell survival, metabolic adaptations, and uncontrolled cell division characters leading to cancer (Subramanian et al., 2005). BRD3 and BRD4 regulate the proto oncogenic activities of MYC (Padmanabhan et al., 2016).

**FIGURE 1:**
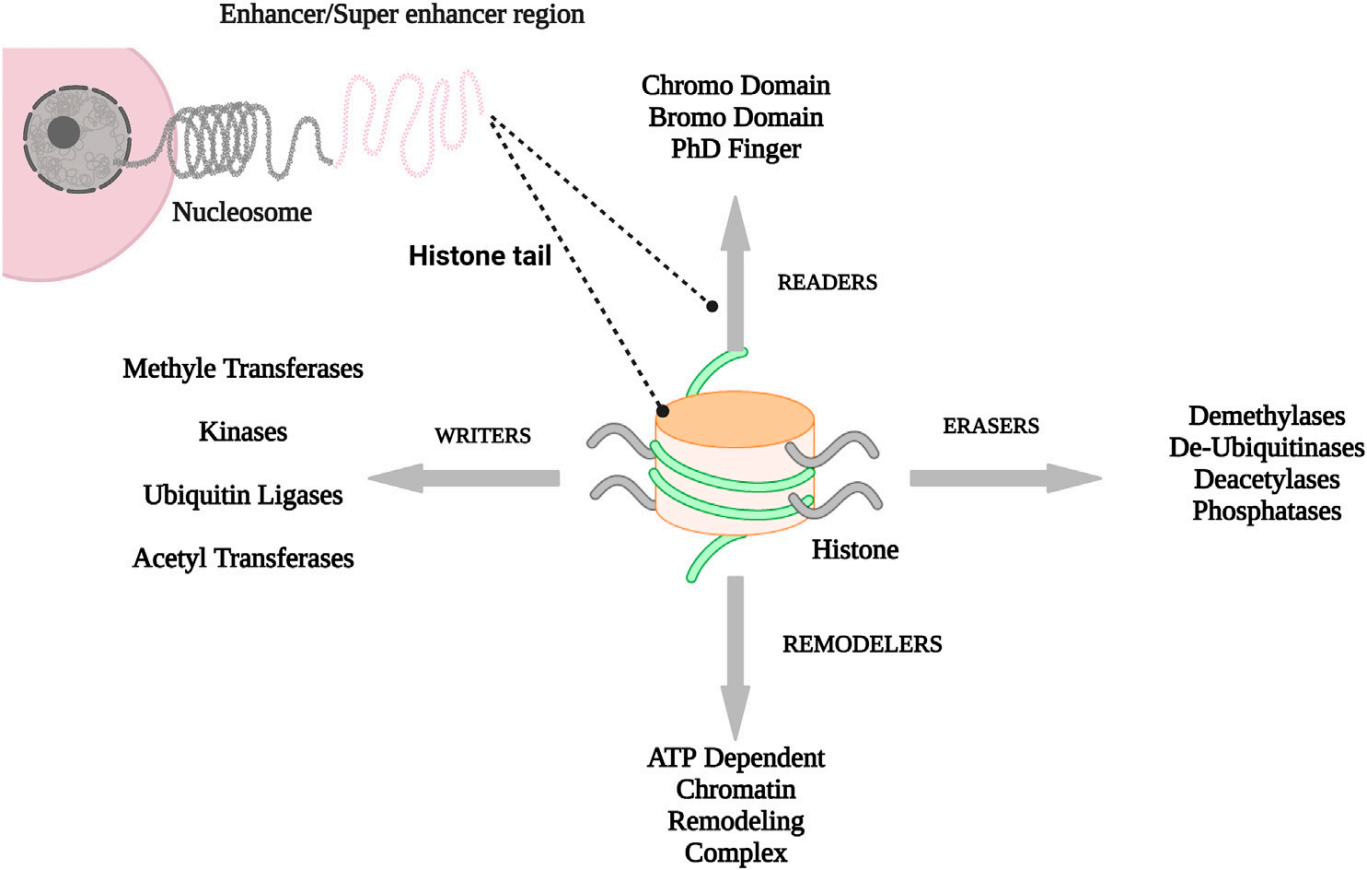
A brief overview of gene transcription: how genetic information in cell transfer from nucleus (right part of figure) to histone tail leading to histone posttranslational modification (PTMs) (left part of figure). Created with BioRender.com.

**FIGURE 2:**
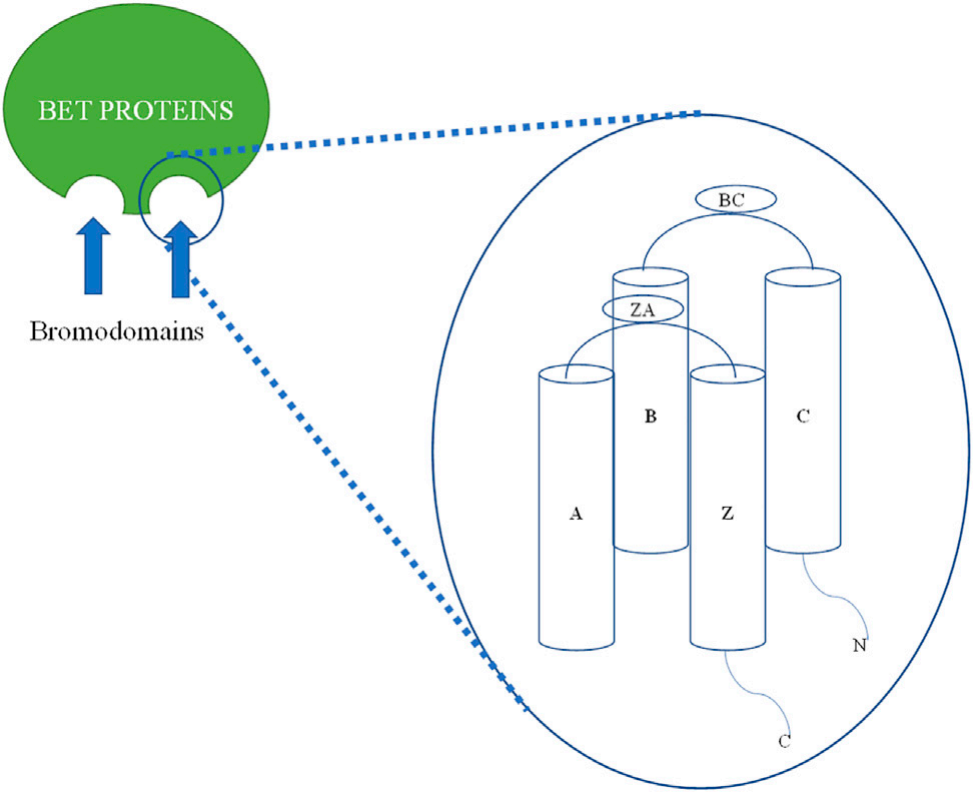
Two-dimensional (2D) structural diagram of BET bromodomain (BRD2, BRD3, and BRD4) proteins: All BET bromodomain proteins have common four alpha-helical bundles named as αZ, αA, αB, and αC. αB and αC are connected by a BC loop, and a ZA loop connects αZ, αA helices (based on x-ray crystal structure).

**FIGURE 3:**
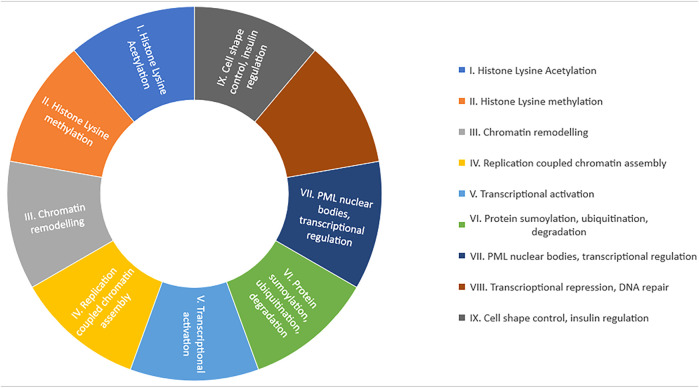
Summery of functional classification of human bromodomain protein.

**FIGURE 4:**
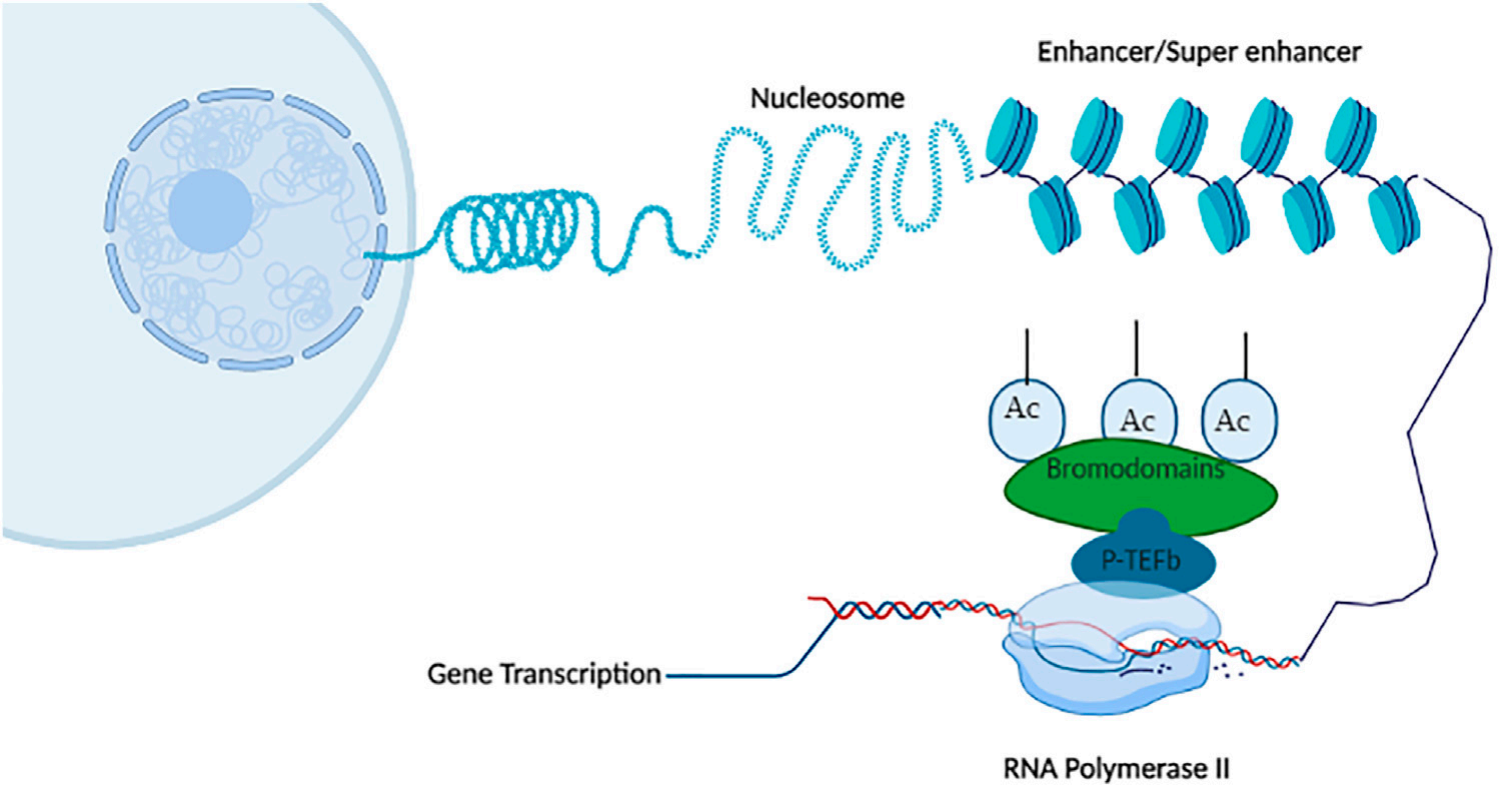
P-TEFb complex-associated gene transcription: BET bromodomain proteins (BRD4, BRDT) recruit positive transcription elongation factor b (P-TEFb) transcription factor at RNA polymerase II leading to phosphorylation of RNA Pol II, which results in gene transcription and elongation. Created with BioRender.com.

Stomal cells present in TME accelerate the expression of cyclin D1 protein. Cyclin D1 expression is regulated by (Figures 1–4) MYC (Tables 1, 2) and NF-κB, and it promotes the synthesis of TNF alpha, CXCL1, CXCL5, CXCL9, CCL2, CXCL12, CCL7, CCL11, and TNF beta-like proinflammatory chemokines and cytokines, which give aggressive behavior to cancer (especially breast cancer). Chronic inflammation causes increase in cyclin D1 proinflammatory chemo and cytokines, which enhance differentiation to CD11b MDSCs (myeloid-derived suppressor cells) from CD34-positive hematopoietic stem cells. MSDCs augment the TME invasion capabilities of cancer. BRD4 mediates transcriptional signaling of cyclin D1 and MSDCs in stomal and inflammatory cells that are associated with tumor progression (Pestell et al., 2017).

**TABLE 1:** Examples of some bromodomain and extraterminal (BET) inhibitors and their BD1 or BD2 binding affinities over counter BD domain.

BD1 affinitive BET inhibitors	BD2 affinitive BET inhibitors
GSK778: 1,000-fold	Apabetalone (RVX-208): 3–40 times
GSK789: >130 times	ABBV-744: >300 times
(+)- JQ1: 45–50 times	GSK620: 300 times
Xanthine derivatives: 10-fold	GSK046: 300 times
Olinone: 100 times	BY27: 5–38 times
Compound MS436: 10-fold	I-BET762: 20-fold

**TABLE 2:** A summary of BET bromodomain protein physiological functions.

BRD2	i. Stimulates E2F1 and E2F2 proteins, which promote the synthesis of proteins needed for the G1/S phase
	ii. BRD2 is expressed in mammary glands, ovaries and testes, uterus, epididymis
	iii. Plays a vital role in neural tube closure and dorsal root completion during embryogenesis
	iv. Pleiotrophin (Ptn) is a neuronal growth factor responsible for neuroprotection, Ptn interrelates with BRD2 and improves cell differentiating and stimulating activities of BRD2 (Padmanabhan et al., 2016)
	v. Promotes gene transcription, elongation, and chromatin
	vi. Involved in epigenetic memory
	vii. Controls the expression of transcriptional regulators, cyclin D1 E and A (Stonestrom et al., 2015)
	viii. Controls the expression of GATA1 (erythroid transcription factor) and function in maturation of erythroid (Stonestrom et al., 2015)
	ix. Plays an important role in neurogenesis and embryogenesis of mammals (Gyuris et al., 2009)
	x. Improves insulin signaling and improve metabolic disorders (Sun et al., 2017)
	xi. Involved in the activation and regulation of NF-κB-mediated inflammatory response (Gallagher et al., 2014)
BRD3	i. Controls the expression of erythroid and megakaryocyte-specific genes
	ii. Transcriptional regulator of cyclin D1, which is vital for the transition of dividing cells from G1 to S phase (Lamonica et al., 2011)
	iii. Possesses properties of nucleosome assembly, a process called “nucleosome chaperone” (Lamonica et al., 2011)
BRDT	i. Involved in spermatogenesis in the male germline, BRDt expression starts, which lasts until the post-meiotic stage (Lamonica et al., 2011)
	ii. Cyclin A1 gene is a regulatory gene in male germ cell lines and is necessary for spermatocytes to enter the first meiotic division. BRDt initiates and controls cyclin A1 expression (Lamonica et al., 2011)
	iii. Performs a transcriptional repressor role or activator role during spermatogenesis (Lamonica et al., 2011)
	iv. C-terminal motif sequences of BRDt and BRD4 have similarities (Morinière et al., 2009)
	v. BRDT is necessary for the recruitment of P-TEFb with BRD4 and is responsible for chromatin remodeling by interacting with hyperacetylated nucleosomes (Bisgrove et al., 2007)
BRD4	i. Plays a pivotal role in the control of cell cycle, embryogenesis, and stabilizing the genome (Wu and Chiang, 2007)
	ii. Recruits P-TEFb, for phosphorylation of RNA polymerase II at transcriptional start sites and persuades transcription elongation (Jang et al., 2005; Bisgrove et al., 2007)
	iii. BRD4 and mediator complex reside in the same enhancer subset, which is called super-enhancer (SE) (Lovén et al., 2013; Filippakopoulos and Knapp, 2014; Bhagwat et al., 2016)
	iv. SEs are crucial for lineage-specific and growth survival-promoting gene transcription (Lovén et al., 2013; Filippakopoulos and Knapp, 2014; Bhagwat et al., 2016)
	v. Plays a role in controling cell cycle regulation as it is critical for the cell to enter into mitosis (Mochizuki et al., 2008; Yang et al., 2008)
	vi. Extraterminal domain (ETD) or extra terminal motif (ETM) of BRD4 enrols transcription activators
	vii. It can ligate to other acetylated proteins also, such as transcription factors (Alpatov et al., 2014)
	viii. BRD4 possesses kinase activity. ETD can attach to SWI-SNF and CHD2, which are responsible for ATP-dependent chromatin remodeling (Rahman et al., 2011; Shi et al., 2013)
	ix. BRD4 is critical from G2 to M transition during the cell cycle (Dey et al., 2000)
	x. BRD4 is critical for neuronal development and arbitrate transcriptional regulation essential for learning behavior and memory (Korb et al., 2015)
	xi. BRD4 recruits E2 at acetylated histone functions, such as a transcriptional regulator in the HPV virus (You et al., 2004; Abbate et al., 2006)
	xii. Presents kinase activity, as BRD4 phosphorylates RNA Pol II resulting in topoisomerase I activation, which facilitates RNA Pol II transition progression and DNA decompaction (Devaiah et al., 2012)
	xiii. BRD4 interacts with histone modifiers and TFs, involved in the differentiation of inflammatory CD4 T lymphocytes to T1 helper cells, such as Th1, Th2, and Th17 cells (Winter et al., 2017)
	xiv. A direct contributor of DNA double-strand break (DSB) repair by the conventional and uncanonical way (Zhao et al., 2000; Zhang et al., 2003)
	xv. Possesses telomere-maintaining properties as it recruits the telomerase and telomerase-associated complexes at short telomers, facilitating, lengthening, and stabilizing the telomers (Zhao et al., 2000; Zhang et al., 2003)

Nuclear factor-kappa B (NF-κB) inflammatory cytokines and peroxisome proliferator-activated receptor gamma (PPAR- γ ), adipocyte differentiation genes, are involved in the cell’s metabolic process. These are regulated by BET proteins, and BETs can activate or repress simultaneously both PPAR- γ and NF-κB genes. Obese women with type 2 diabetes (T2D) and chronic inflammatory diseases are at higher risk of breast cancer, asT2D with obesity mediates the production of IL6, IL7, and CD68 macrophages, such as proinflammatory cytokines, which exacerbate the invasion of metastatic cancerous cells. BET proteins (mainly BRD2), being transcription regulators of inflammatory and proinflammatory genes, induce metabolic breast cancer (Wang et al., 2009; Andrieu et al., 2018).

#### 5.1.1 Roles of BRD4 in carcinogenesis

Aberrant expression of BET proteins leads to cancer, inflammatory diseases, metabolic disorders, and neurodegenerative diseases. BRD4, the most studied protein is dysregulated in numerous diseases, and the major one is cancer. The delocalization of amino acid sequence in BRD4 at the proximal part (which binds to acetylated histone lysine) αB and αC terminals changes the tertiary structure, protein–protein, protein–DNA interaction and conformation. This substitution changes in structure and conformation of BRD4 results in the arbitration of oncogenic characters and leads to tumor genesis, metastasis, and tumor progression. BRD4 regulates and maintains MYC expression in metastasis, and this shows that the BET is used for MYC transcription target strategy (Lin et al., 2012).

#### 5.1.2 BRD4 and hematopoietic cancers

BRD4 activates and keeps the MYC expression constant in hematopoietic cancers. AML patients report abnormal transcription elongation and MYC high expression (Zuber et al., 2011). In DLBCL (diffused large B-cell lymphoma) patients, 32% showed overexpression of MYC. BL is also characterized by increase in MYC expression in B cells leading to lymphomagenesis (Mertz et al., 2011; Horn et al., 2013). NPM1 (nucleophosmin) is a nuclear protein that is involved in ribosomal assembly and regulation of the ARF-p53 cascade. It can move back and forth in the cytoplasm to perform normal cellular functions. NPM1 is reported to be highly expressed in AML. About 35% of AML patients have NPM1 high protein, which is a distinctive feature of AML from other types of hematopoietic cancers (Döhner et al., 2005). Normally, NPM1 interaction with BRD4 in the nucleus activates the P-TEFb repressor component HEXIM, which blocks the transcription elongation mediated by BRD4. In AML, BRD4-facilitated transcription elongation repression is reduced due to mutation of cytoplasmic confined NPM1 gene, which results in overexpression of tumor progressive MYC gene along with BCL2 gene (Dawson et al., 2014).

Methyltransferase lysine 2A or lysine methyltransferase 2A (KMT2A) gene regulates transcription. It is involved in chromosomal translocation in about 10% of hematopoietic myelomas. About 22% of AML children and 5% of adult patients have chromosomal translocation due to KMT2A (Thirman et al., 1993; Krivtsov and Armstrong, 2007). KMT2A fusion with elongation transcriptional regulators (e.g., SE) results in dysregulation of transcription in the development of leukemia. BRD3 and BRD4 facilitate the localization of KMT2A and, thus, help in transcription and chromosomal translocation (Dawson et al., 2011).

#### 5.1.3 NUT carcinoma

NUT carcinoma (NC) or NUT midline carcinoma is a rare category of squamous carcinoma, which affect almost all ages but mostly teen and young aged ones. NC origin cells and anatomical position are not well known because when it is diagnosed, it is spread to many parts of the body (Bauer et al., 2012; Chau et al., 2016). It is one of the highly aggressive types of cancer but is poorly diagnosed (Ball et al., 2012). Some studies indicate its origin in the head, neck, and thorax as it stereotypically ascends from the upper airway midlines (French, 2012), but it also exists in the pancreas, kidneys, lungs, adrenal glands, soft bone tissues, salivary glands, etc. (Ziai et al., 2010). Genetically, NC arises from translocation of nuclear protein in testis (NUT, which is specifically expressed in testis) gene at chromosomal 15 arms with BRD4 on chromosome 19q13.1 [t (15; 9) (q14; p13.1)] leading to the production of a BRD4–NUT fusion protein. Sometimes, the NUT gene can make a fusion with BRD3 at chromosome 9q34.2 [t (15; 9) (q14; q34.2)], resulting in the BRD3–NUT fusion protein (French et al., 2004; French et al., 2008). The BRD4–NUT fusion protein reduces BRD4 isoform expression and potentiates the propensity of BRD4–NUT that influences the free BRD4 to perform cellular differentiation functions, thus, accelerating oncogenic behavior of NM cells (Padmanabhan et al., 2016). Statistical data of NC patients show that 88% have BET–NUT fusion protein, in which 71% are BRD–NUT, while 14% are BRD3–NUT fusion (Floyd et al., 2013).

#### 5.1.4 Prostate cancer

Prostate cancer (PC) reports state that there are more than 300,000 cases per year in the United States, and it is a common cause of cancer of men in Europe and America. BRD4, being a regulator of chromatin remodeling, maintains chromatin and nucleosome compaction. Overexpression of BRD4 causes loss of chromatin compaction, nucleosome expulsion, which increases the chromatin accessibility and co-activates androgen receptor (AR) (Braadland and Urbanucci, 2019). BRD4 overexpression degrades AR and deregulates its expression, leading to prostate cancer or castration-resistant prostate development (Urbanucci et al., 2017). Castration-resistant prostate cancer (CRPC) is a progressive type of PC that occurs when resistance develops to conservative androgen deprivation therapy (ADP). ADP develops by multiple factors including a mutation in AR, gene amplification, and destruction of AR expression. BRD4, as transcription regulator along with lysine methyltransferase 2A, performs a transcription activator role and helps in PC advancement to CRPC. BRD4 is a basis of CRPC cell migration and invasion through AHNAK (protein that potentiates metastasis in a variety of cancers) transcription (Urbanucci and Mills, 2018; Shafran et al., 2019).

#### 5.1.5 Inflammation and cancer

Chronic inflammation induced by either infections or metabolic reasons can be a stage for cancer development, as chronic inflammation can transform the normal body cells to neoplastic and metastatic cells and initiate tumor genesis (Calle et al., 2003; Dalmas et al., 2014). MYC is upregulated in inflammatory conditions that associates tumorgenesis with inflammation (Howe et al., 2013). Cancerous cell development requires the cells to have SASP (secretory-associated senescent phenotype), which recruits BRD4 at enhancer regions. BRD4 recruitment increases the expression of BMP2, IL-1α, inhibin βA, IL-1β, and IL-8 SASP factors which induce malignancies (Tasdemir et al., 2016). Octamer-binding transcription factor 2 (Oct2) is vital for B-cell definite gene expression. BRD4 controls B-cell expression as it interrelates with Oct2. BRD4 can also bind to acetylated non-histone lysine, one such important interaction is the BRD4-NF-κB. The p65/RELA factor of NF-κB is acetylated at lysine 310 b y BRD4, which activates NF-κB. NF-κB activation recruit P-TEFb active factor CDK9 through BRD4, which leads to NF-κB-marked gene stimulation (Huang et al., 2009). Enhancer RNA (eRNA) synthesis and chromatin acetylation at histone 3 lysine 4 arbitrate NF-κB-dependent proinflammatory response. Enhancers regulate the transcription of eRNA, which indicates the NF-κB part in proinflammatory cascade as modulator (Kaikkonen et al., 2013; Hah et al., 2015). In cancer development, BRD4 linkage to NF-κB prevents RELA ubiquitylation, which then blocks RELA degradation through proteasome. This proteasome-mediated degradation inhibition of RELA results in NF-κB activation constantly leading to malignant cell proliferation (Bayarsaihan, 2011).

### 5.2 Role of BRD4 in asthmatic airway remodeling

Allergic asthma (AA) is a lingering illness of airway inflammation caused by aeroallergen exposure of acute exacerbation or infections. Aeroallergens excite Toll-like receptor (TLR) signaling, which is a key factor in the pathology of AA, that originates from the source of inflammation and oxidative stress injury. The supercoiling of chromosomes at super-enhancer regions are accountable for gene expression. In TLR signaling or inflammation, repositioning of BRD4 at super-enhancer stations occur, which increases inflammatory gene expression (Whyte et al., 2013). TLRs persuade reactive oxygen stress (ROS), which provokes DNA damage and acts as a messenger for the release of growth factors and inflammatory cytokines. These events amend normal gene expression and become the basis of airway remodeling (Choudhary et al., 2016). OGG1 (8-oxoguanine DNA glycosylates) is an epigenetic pleiotropic signal protein involved in DDR and responsible for innate immunity (Pan et al., 2016). ROS provokes oxidation of guanine base to formulate 8-oxoG (7,8 dihydro-8-oxoguanine). OGG1 identifies 8-oxoG at promoter sections and binds to BRD4 mediator TF NF-κB and potentiates inflammatory mediator gene subset recruitment. BRD4 facilitated recruitment of inflammatory gene monitors CXCL2 (C-X-C motif ligand 2), such as neutrophilic chemokine expression and cytokine production, which are the main culprit in pollen allergy and TNF-responsive leukocyte inflammation (Bacsi et al., 2013; Hao et al., 2018).

### 5.3 Role of BRD4 in cardiac injury

BRD4 is one of the main culprits of cardiac injuries, including cardiac hypertrophy, infarction congestive heart failure, and vascular smooth muscles remodeling. Sun et al. established a rat myocardial infarction model (MIM) to reveal the BET protein role. The results demonstrated that protein levels, as well as mRNA levels of BRD2 and BRD4, were elevated in MIM rats compared with the sham group (Sun et al., 2015). ROS generation is a major contributor to the pathogenesis of cardiac diseases including cardiac hypertrophy. A study by Zhu et al. indicated the upregulation of BRD4 protein expression in pressure overload-induced cardiac myocytes and fibroblasts. Oxidative stress and ROS generation provoke the BRD4 levels and associated cardiac remodeling, hypertrophy, and fibrosis (Zhu et al., 2020). High glucose induction to H9C2 cells (a cell line to study cardiac pathogenesis *in vitro*) causes cardiac hypertrophy and increases the expression of BRD4, which was further confirmed by the *in vivo* model of diabetic rats (Wang et al., 2019).

### 5.4 Bromodomain and extraterminal proteins and renal diseases

Chronic kidney disease (CKD) is defined by alteration or loss of nephron structure and functions. The pathological condition is worsened by 1) altered intracellular mechanisms, such as inflammatory cascade, and 2) factors like diabetes, obesity, non-alcoholic fatty liver diseases, and hypertension that trigger kidney impairment and lead to renal fibrosis and end-stage renal diseases (ESRD). Studies indicate that tainted pathological and environmental factors in CKD are linked to epigenetic alterations and modifications. Those epigenetic alterations, which are linked to kidney pathologies, are DNA methylation, changes in miRNA, and histone modifications. BET proteins bind to transcriptional factor NF-κB, which causes overexpression of CCL2 mRNA and proinflammatory gene mediator, and sustain inflammation in CKD (Keating and El-Osta, 2013; Morgado-Pascual et al., 2018).

In renal pathology, enhancer regions of inflammatory genes, such as CCL-2 and IL-6, bind to BRD4 at acetylated histone (Suarez-Alvarez et al., 2017). BRD4, as TF, regulates and implicates inflammatory CD4 cell differentiation into Th cells (Th1, Th2, and Th17). The important cells involved in immune, non-immune, and chronic renal diseases are Th 17 cells and their active IL-17A cytokine. BRD4 binds to CNS2, which controls IL-17 transcription, thus, participates openly in Th17 transcription. Cardiovascular events are one of the main death causes in CKD patients. BRD4 is an important gene transcription activator in cardiac hypertrophy and heart failure (Wang et al., 2019).

## 6 Approaches to inhibit bromodomain and extraterminal proteins

Developing BET inhibitors for cancer and other epigenetic diseases is a challenging and hot topic in drug development researchers and laboratories. There are various approaches to target BET proteins; these include.

i. Development of PROTAC (proteolytic targeting chimaera) compounds, e.g., dBETi, MZ1, ARV-825 (DeMars et al., 2019; Lim et al., 2019).
ii. AZD5153, AZD4320, etc., belong to the bivalent class of BETi, and they ligate to both domains with almost the same affinity. It has the advantage of displacing the whole BET proteins (BRD4) by possessing higher potency than monovalent BETi at relatively lower concentrations (Rhyasen et al., 2016).
iii. BET inhibitors bind covalently to the bromodomain domains of BETs. These BETi provide BET inhibition at a relatively lower dose and offer to sustain pharmacological effects. ZEN-3219 and ZEN-3862 are covalent BETi (Kharenko et al., 2018).
iv. Noncovalent BETi that interact specifically to only one domain (either BD1or BD2). JQ1, OTX015, I-BET762, etc., are some examples of monovalent BETi, which ligate to BD1, while Apabatlon binds to BD2 (Dawson et al., 2011; Ferri et al., 2016).

## 7 The selectivity of noncovalent bromodomain and extraterminal inhibitor bromodomain binding

Noncovalent BETi are mainly divided into two categories.

i. Nonselective: These inhibitors ligate to both domains of BET proteins, i.e., BD1 and BD2. Initially, the inhibitors developed were nonselective, but due to their toxic activity, researchers projected developing selective BETi, which helps to observe their unique and individual activity and biological response.
ii. Selective BETi: they are further subcategorized according to their affinities toward binding to BD1 or BD2
iia BD1 affinitive:
• GSK789 has a 1,000-fold higher affinity for BD1 than BD2 (Ray et al., 2020).
• GSK778 exhibits >130-fold BD1 selectivity over BD2 due to BD1 Asp144/His433 displacement (Kharenko et al., 2016).
• (+)-JQ1 has 45–50 times more binding capabilities to BD1 compared with BD2 (Chen et al., 2021).
• Xanthine derivatives bind to BD1 with 10 times the affinity (Gilan et al., 2020).
• Olinone shows 100-fold more affinity toward BD1 due to mobile hydrogen bonds in the BC and ZA loop (Gilan et al., 2020).
• MS436 forms a hydrogen bond with the phenyl group of BD1’s Tyr97 amino acid and has 10 times the selectivity of BD2 (Fu et al., 2021).
iib BD2 affinitive:
• RVX-208 (Apabetalone), which is a BD2-selective BETi showing 30- to 40-fold high affinity over BD1 (Picaud et al., 2013). Apabetalone is now in clinical phase 3 for CVD (Kharenko et al., 2016; Ray et al., 2020).
• ABBV-744 is another BD2-selective BETi that possesses >300-fold more binding affinity over BD1 (Chen et al., 2021).
• The improved pharmacokinetic properties of BD2-selective BETi are leading to the discovery and development of more and more BD2-selective BETi, examples are GSK620 and GSK046 (more than 300-fold BD2 ligation affinity over BD1) (Gilan et al., 2020).
• BY27 (5- to 38-fold BD2 binding selectivity compared with BD1) (Chen et al., 2019).
• I-BET762 is 20 times BD2 specific than BD1 (Fu et al., 2021).

## 8 Conclusion

Bromodomains (BRDs) and extraterminals (ETs) are epigenetic proteins that function as readers of acetylated histone. There are four types of such epigenetic readers, namely, BRD2, BRD3, BRDT, and BRD4. They are responsible for the regulation and progression of cell cycle, spermatogenesis, embryogenesis, neurogenesis, DNA strand break repair, and are involved in learning and growth, etc. Based on their cellular functions, these acetylated reader proteins are classified into nine groups. Besides these, they are also a major contributor to many pathological conditions, such as cancer (hematopoietic cancer, NUT carcinoma, prostate cancer, etc.), renal diseases, diabetes, inflammation, and cardiac injury. This review sheds a brief light on biochemistry, physiological functions, pathophysiological roles, and their downstream and upstream target proteins. The review presents a comprehensive understanding of BET bromodomains and current and future approaches to counter BET bromodomain-associated diseases. There is a comprehensive description of BETi currently in biological research (GSK 778 and GSK789) and clinical trials (JQ1, RVX-208). Although, nowadays, BETi are developed and studied according to their affinities toward BD1 or BD2 domain, still, they are nonspecific in their target and mechanism. As most of the BETi are nonspecific in their mechanism. BRTi are extremely potent, with a very narrow therapeutic window. So, there is a need for further study on BET bromodomain proteins and to develop such BETi that are specific for a particular pathological condition and have the least side effects.

## Glossary

HM: Histone modifications
KAc: Acetyl lysine
Gln: Glutamine
Asp: Aspartic acid
Lle (L-Ie): Isoleucine
al: Valine
His: Histidine
BRPF1/2/3: Bromodomain and PHD finger-containing protein
MOZ: Monocytic leukemia Zing finger protein
SMARCA2/4 SWI/SNF Related: Matrix associated, actin-dependent regulator of chromatin, subfamily A (Member 2, 4)
SWI/SNF: Switch/sucrose nonfermentable
ISWI: Imitation SWI
TIF1: Transcriptional intermediary factor 1
PML-NB: Subnuclear bodies of promyelocytic leukemia
WD: repeat protein family containing 40 amino acids especially tryptophan and aspartic acid end chain
CTD: C-terminal domain
P-TEFb: Positive transcription elongation factor b
7SK: Small nuclear RNA is a multifunctional transcriptional regulatory RNA that controls the nuclear activity of the P-TEFb
DEAF-1: (Deformed epidermal autoregulatory factor-1)
snRNA: 7SK small nuclear RNA
CDK9: Cyclin-dependent kinase 9
SPA-1: Signal-induced proliferation-associated gene-1
HEXIM: Hexamethylene bisacetamide inducible 1
TWIST: Twist-related protein 1
RING3: Really interesting new gene 3
FSHRG1: Female sterile homeotic-related gene 1
TRIM 28: Tripartite motif containing 28 proteins
PRMT5: Protein arginine methyltransferase 5

## References

[B1] Abbate E. A. Voitenleitner C. Botchan M. R. (2006). Structure of the Papillomavirus DNA-Tethering Complex E2:Brd4 and a Peptide that Ablates HPV Chromosomal Association. Mol. Cell 24 (6), 877–889. 10.1016/j.molcel.2006.11.002 17189190

[B2] Allfrey V. G. Faulkner R. Mirsky A. E. (1964). Acetylation and Methylation of Histones and Their Possible Role in the Regulation of Rna Synthesis. Proc. Natl. Acad. Sci. U S A. 51 (5), 786–794. 10.1073/pnas.51.5.786 14172992 PMC300163

[B3] Allis C. D. Jenuwein T. (2016). The Molecular Hallmarks of Epigenetic Control. Nat. Rev. Genet. 17 (8), 487–500. 10.1038/nrg.2016.59 27346641

[B4] Alpatov R. Lesch B. J. Nakamoto-Kinoshita M. Blanco A. Chen S. Stützer A. (2014). A Chromatin-dependent Role of the Fragile X Mental Retardation Protein FMRP in the DNA Damage Response. Cell 157 (4), 869–881. 10.1016/j.cell.2014.03.040 24813610 PMC4038154

[B5] Andrieu G. P. Shafran J. S. Deeney J. T. Bharadwaj K. R. Rangarajan A. Denis G. V. (2018). BET Proteins in Abnormal Metabolism, Inflammation, and the Breast Cancer Microenvironment. J. Leukoc. Biol. 104 (2), 265–274. 10.1002/JLB.5RI0917-380RR 29493812 PMC6134394

[B6] Angrand P. O. Apiou F. Stewart A. F. Dutrillaux B. Losson R. Chambon P. (2001). NSD3, a New SET Domain-Containing Gene, Maps to 8p12 and Is Amplified in Human Breast Cancer Cell Lines. Genomics 74 (1), 79–88. 10.1006/geno.2001.6524 11374904

[B7] Bacsi A. Aguilera-Aguirre L. Szczesny B. Radak Z. Hazra T. K. Sur S. (2013). Down-regulation of 8-oxoguanine DNA Glycosylase 1 Expression in the Airway Epithelium Ameliorates Allergic Lung Inflammation. DNA Repair (Amst) 12 (1), 18–26. 10.1016/j.dnarep.2012.10.002 23127499 PMC3678389

[B8] Ball A. Bromley A. Glaze S. French C. A. Ghatage P. Köbel M. (2012). A Rare Case of NUT Midline Carcinoma. Gynecol. Oncol. Case Rep. 3, 1–3. 10.1016/j.gynor.2012.09.004 24371650 PMC3862205

[B9] Bannister A. J. Kouzarides T. (2011). Regulation of Chromatin by Histone Modifications. Cell Res. 21 (3), 381–395. 10.1038/cr.2011.22 21321607 PMC3193420

[B10] Barrero M. J. (2017). Epigenetic Strategies to Boost Cancer Immunotherapies. Int. J. Mol. Sci. 18 (6), 1. 10.3390/ijms18061108 PMC5485932 28545238

[B11] Bauer D. E. Mitchell C. M. Strait K. M. Lathan C. S. Stelow E. B. Lüer S. C. (2012). Clinicopathologic Features and Long-Term Outcomes of NUT Midline Carcinoma. Clin. Cancer Res. 18 (20), 5773–5779. 10.1158/1078-0432.CCR-12-1153 22896655 PMC3473162

[B12] Bayarsaihan D. (2011). Epigenetic Mechanisms in Inflammation. J. Dent Res. 90 (1), 9–17. 10.1177/0022034510378683 21178119 PMC3144097

[B13] Belkina A. C. Nikolajczyk B. S. Denis G. V. (2013). BET Protein Function Is Required for Inflammation: Brd2 Genetic Disruption and BET Inhibitor JQ1 Impair Mouse Macrophage Inflammatory Responses. J. Immunol. 190 (7), 3670–3678. 10.4049/jimmunol.1202838 23420887 PMC3608815

[B14] Bhagwat A. S. Roe J. S. Mok B. Y. L. Hohmann A. F. Shi J. Vakoc C. R. (2016). BET Bromodomain Inhibition Releases the Mediator Complex from Select Cis-Regulatory Elements. Cell Rep. 15 (3), 519–530. 10.1016/j.celrep.2016.03.054 27068464 PMC4838499

[B15] Bird A. P. (1986). CpG-rich Islands and the Function of DNA Methylation. Nature 321 (6067), 209–213. 10.1038/321209a0 2423876

[B16] Bisgrove D. A. Mahmoudi T. Henklein P. Verdin E. (2007). Conserved P-TEFb-Interacting Domain of BRD4 Inhibits HIV Transcription. Proc. Natl. Acad. Sci. U S A. 104 (34), 13690–13695. 10.1073/pnas.0705053104 17690245 PMC1959443

[B17] Bottomley M. J. Collard M. W. Huggenvik J. I. Liu Z. Gibson T. J. Sattler M. (2001). The SAND Domain Structure Defines a Novel DNA-Binding Fold in Transcriptional Regulation. Nat. Struct. Biol. 8 (7), 626–633. 10.1038/89675 11427895

[B18] Braadland P. R. Urbanucci A. (2019). Chromatin Reprogramming as an Adaptation Mechanism in Advanced Prostate Cancer. Endocr. Relat. Cancer 26 (4), R211–r235. 10.1530/ERC-18-0579 30844748

[B19] Calle E. E. Rodriguez C. Walker-Thurmond K. Thun M. J. (2003). Overweight, Obesity, and Mortality from Cancer in a Prospectively Studied Cohort of U.S. Adults. N. Engl. J. Med. 348 (17), 1625–1638. 10.1056/NEJMoa021423 12711737

[B20] Chau N. G. Hurwitz S. Mitchell C. M. Aserlind A. Grunfeld N. Kaplan L. (2016). Intensive Treatment and Survival Outcomes in NUT Midline Carcinoma of the Head and Neck. Cancer 122 (23), 3632–3640. 10.1002/cncr.30242 27509377 PMC5361614

[B21] Chen D. Lu T. Yan Z. Lu W. Zhou F. Lyu X. (2019). Discovery, Structural Insight, and Bioactivities of BY27 as a Selective Inhibitor of the Second Bromodomains of BET Proteins. Eur. J. Med. Chem. 182, 111633. 10.1016/j.ejmech.2019.111633 31461688

[B22] Chen J. Li Y. Zhang J. Zhang M. Wei A. Liu H. (2021). Discovery of Selective HDAC/BRD4 Dual Inhibitors as Epigenetic Probes. Eur. J. Med. Chem. 209, 112868. 10.1016/j.ejmech.2020.112868 33077265

[B23] Choudhary C. Kumar C. Gnad F. Nielsen M. L. Rehman M. Walther T. C. (2009). Lysine Acetylation Targets Protein Complexes and Co-regulates Major Cellular Functions. Science 325 (5942), 834–840. 10.1126/science.1175371 19608861

[B24] Choudhary S. Boldogh I. Brasier A. R. (2016). Inside-Out Signaling Pathways from Nuclear Reactive Oxygen Species Control Pulmonary Innate Immunity. J. Innate Immun. 8 (2), 143–155. 10.1159/000442254 26756522 PMC4801701

[B25] Dalmas E. Venteclef N. Caer C. Poitou C. Cremer I. Aron-Wisnewsky J. (2014). T Cell-Derived IL-22 Amplifies IL-1β-driven Inflammation in Human Adipose Tissue: Relevance to Obesity and Type 2 Diabetes. Diabetes 63 (6), 1966–1977. 10.2337/db13-1511 24520123

[B26] Dang C. V. (2012). MYC on the Path to Cancer. Cell 149 (1), 22–35. 10.1016/j.cell.2012.03.003 22464321 PMC3345192

[B27] Dawson M. A. Gudgin E. J. Horton S. J. Giotopoulos G. Meduri E. Robson S. (2014). Recurrent Mutations, Including NPM1c, Activate a BRD4-dependent Core Transcriptional Program in Acute Myeloid Leukemia. Leukemia 28 (2), 311–320. 10.1038/leu.2013.338 24220271 PMC3918873

[B28] Dawson M. A. Prinjha R. K. Dittmann A. Giotopoulos G. Bantscheff M. Chan W. I. (2011). Inhibition of BET Recruitment to Chromatin as an Effective Treatment for MLL-Fusion Leukaemia. Nature 478 (7370), 529–533. 10.1038/nature10509 21964340 PMC3679520

[B29] Deeney J. T. Belkina A. C. Shirihai O. S. Corkey B. E. Denis G. V. (2016). BET Bromodomain Proteins Brd2, Brd3 and Brd4 Selectively Regulate Metabolic Pathways in the Pancreatic β-Cell. PLoS One 11 (3), e0151329. 10.1371/journal.pone.0151329 27008626 PMC4805167

[B30] DeMars K. M. Yang C. Candelario-Jalil E. (2019). Neuroprotective Effects of Targeting BET Proteins for Degradation with dBET1 in Aged Mice Subjected to Ischemic Stroke. Neurochem. Int. 127, 94–102. 10.1016/j.neuint.2019.03.004 30872008

[B31] Denis G. V. Green M. R. (1996). A Novel, Mitogen-Activated Nuclear Kinase Is Related to a Drosophila Developmental Regulator. Genes Dev. 10 (3), 261–271. 10.1101/gad.10.3.261 8595877

[B32] Denis G. V. McComb M. E. Faller D. V. Sinha A. Romesser P. B. Costello C. E. (2006). Identification of Transcription Complexes that Contain the Double Bromodomain Protein Brd2 and Chromatin Remodeling Machines. J. Proteome Res. 5 (3), 502–511. 10.1021/pr050430u 16512664 PMC2823066

[B33] Denis G. V. Vaziri C. Guo N. Faller D. V. (2000). RING3 Kinase Transactivates Promoters of Cell Cycle Regulatory Genes through E2F. Cell Growth Differ 11 (8), 417–424. 10965846 PMC3968681

[B34] Devaiah B. N. Case-Borden C. Gegonne A. Hsu C. H. Chen Q. Meerzaman D. (2016). BRD4 Is a Histone Acetyltransferase that Evicts Nucleosomes from Chromatin. Nat. Struct. Mol. Biol. 23 (6), 540–548. 10.1038/nsmb.3228 27159561 PMC4899182

[B35] Devaiah B. N. Lewis B. A. Cherman N. Hewitt M. C. Albrecht B. K. Robey P. G. (2012). BRD4 Is an Atypical Kinase that Phosphorylates Serine2 of the RNA Polymerase II Carboxy-Terminal Domain. Proc. Natl. Acad. Sci. U S A. 109 (18), 6927–6932. 10.1073/pnas.1120422109 22509028 PMC3345009

[B36] Dey A. Ellenberg J. Farina A. Coleman A. E. Maruyama T. Sciortino S. (2000). A Bromodomain Protein, MCAP, Associates with Mitotic Chromosomes and Affects G(2)-to-M Transition. Mol. Cell Biol. 20 (17), 6537–6549. 10.1128/mcb.20.17.6537-6549.2000 10938129 PMC86127

[B37] Dey A. Nishiyama A. Karpova T. McNally J. Ozato K. (2009). Brd4 marks Select Genes on Mitotic Chromatin and Directs Postmitotic Transcription. Mol. Biol. Cell 20 (23), 4899–4909. 10.1091/mbc.e09-05-0380 19812244 PMC2785733

[B38] Dhalluin C. Carlson J. E. Zeng L. He C. Aggarwal A. K. Zhou M. M. (1999). Structure and Ligand of a Histone Acetyltransferase Bromodomain. Nature 399 (6735), 491–496. 10.1038/20974 10365964

[B39] Di Micco R. Fontanals-Cirera B. Low V. Ntziachristos P. Yuen S. K. Lovell C. D. (2014). Control of Embryonic Stem Cell Identity by BRD4-dependent Transcriptional Elongation of Super-enhancer-associated Pluripotency Genes. Cell Rep. 9 (1), 234–247. 10.1016/j.celrep.2014.08.055 25263550 PMC4317728

[B40] Döhner K. Schlenk R. F. Habdank M. Scholl C. Rücker F. G. Corbacioglu A. (2005). Mutant Nucleophosmin (NPM1) Predicts Favorable Prognosis in Younger Adults with Acute Myeloid Leukemia and normal Cytogenetics: Interaction with Other Gene Mutations. Blood 106 (12), 3740–3746. 10.1182/blood-2005-05-2164 16051734

[B41] Ferri E. Petosa C. McKenna C. E. (2016). Bromodomains: Structure, Function and Pharmacology of Inhibition. Biochem. Pharmacol. 106, 1–18. 10.1016/j.bcp.2015.12.005 26707800

[B42] Filippakopoulos P. Knapp S. (2014). Targeting Bromodomains: Epigenetic Readers of Lysine Acetylation. Nat. Rev. Drug Discov. 13 (5), 337–356. 10.1038/nrd4286 24751816

[B43] Filippakopoulos P. Picaud S. Mangos M. Keates T. Lambert J. P. Barsyte-Lovejoy D. (2012). Histone Recognition and Large-Scale Structural Analysis of the Human Bromodomain Family. Cell 149 (1), 214–231. 10.1016/j.cell.2012.02.013 22464331 PMC3326523

[B44] Floyd S. R. Pacold M. E. Huang Q. Clarke S. M. Lam F. C. Cannell I. G. (2013). The Bromodomain Protein Brd4 Insulates Chromatin from DNA Damage Signalling. Nature 498 (7453), 246–250. 10.1038/nature12147 23728299 PMC3683358

[B45] Frank S. R. Parisi T. Taubert S. Fernandez P. Fuchs M. Chan H. M. (2003). MYC Recruits the TIP60 Histone Acetyltransferase Complex to Chromatin. EMBO Rep. 4 (6), 575–580. 10.1038/sj.embor.embor861 12776177 PMC1319201

[B46] French C. A. Kutok J. L. Faquin W. C. Toretsky J. A. Antonescu C. R. Griffin C. A. (2004). Midline Carcinoma of Children and Young Adults with NUT Rearrangement. J. Clin. Oncol. 22 (20), 4135–4139. 10.1200/JCO.2004.02.107 15483023

[B47] French C. A. (2012). Pathogenesis of NUT Midline Carcinoma. Annu. Rev. Pathol. 7, 247–265. 10.1146/annurev-pathol-011811-132438 22017582

[B48] French C. A. Ramirez C. L. Kolmakova J. Hickman T. T. Cameron M. J. Thyne M. E. (2008). BRD-NUT Oncoproteins: a Family of Closely Related Nuclear Proteins that Block Epithelial Differentiation and Maintain the Growth of Carcinoma Cells. Oncogene 27 (15), 2237–2242. 10.1038/sj.onc.1210852 17934517

[B49] Fu Y. Zhang Y. Sun H. (2021). Progress in the Development of Domain Selective Inhibitors of the Bromo and Extra Terminal Domain Family (BET) Proteins. Eur. J. Med. Chem. 226, 113853. 10.1016/j.ejmech.2021.113853 34547507

[B50] Fujinaga K. (2020). P-TEFb as A Promising Therapeutic Target. Molecules 25 (4), 1. 10.3390/molecules25040838 PMC7070488 32075058

[B51] Gallagher S. J. Mijatov B. Gunatilake D. Gowrishankar K. Tiffen J. James W. (2014). Control of NF-kB Activity in Human Melanoma by Bromodomain and Extra-terminal Protein Inhibitor I-Bet151. Pigment Cell Melanoma Res. 27 (6), 1126–1137. 10.1111/pcmr.12282 24924589

[B52] Gamsjaeger R. Webb S. R. Lamonica J. M. Billin A. Blobel G. A. Mackay J. P. (2011). Structural Basis and Specificity of Acetylated Transcription Factor GATA1 Recognition by BET Family Bromodomain Protein Brd3. Mol. Cell Biol. 31 (13), 2632–2640. 10.1128/MCB.05413-11 21555453 PMC3133386

[B53] Gaucher J. Boussouar F. Montellier E. Curtet S. Buchou T. Bertrand S. (2012). Bromodomain-dependent Stage-specific Male Genome Programming by Brdt. Embo j 31 (19), 3809–3820. 10.1038/emboj.2012.233 22922464 PMC3463845

[B54] Gilan O. Rioja I. Knezevic K. Bell M. J. Yeung M. M. Harker N. R. (2020). Selective Targeting of BD1 and BD2 of the BET Proteins in Cancer and Immunoinflammation. Science 368 (6489), 387–394. 10.1126/science.aaz8455 32193360 PMC7610820

[B55] Grivennikov S. I. Greten F. R. Karin M. (2010). Immunity, Inflammation, and Cancer. Cell 140 (6), 883–899. 10.1016/j.cell.2010.01.025 20303878 PMC2866629

[B56] Gyuris A. Donovan D. J. Seymour K. A. Lovasco L. A. Smilowitz N. R. Halperin A. L. (2009). The Chromatin-Targeting Protein Brd2 Is Required for Neural Tube Closure and Embryogenesis. Biochim. Biophys. Acta 1789 (5), 413–421. 10.1016/j.bbagrm.2009.03.005 19362612 PMC2740724

[B57] Hah N. Benner C. Chong L. W. Yu R. T. Downes M. Evans R. M. (2015). Inflammation-sensitive Super Enhancers Form Domains of Coordinately Regulated Enhancer RNAs. Proc. Natl. Acad. Sci. U S A. 112 (3), E297–E302. 10.1073/pnas.1424028112 25564661 PMC4311831

[B58] Hao W. Qi T. Pan L. Wang R. Zhu B. Aguilera-Aguirre L. (2018). Effects of the Stimuli-dependent Enrichment of 8-oxoguanine DNA Glycosylase1 on Chromatinized DNA. Redox Biol. 18, 43–53. 10.1016/j.redox.2018.06.002 29940424 PMC6019822

[B59] Helai P. M. Olena B. Caretha L. C. (2019). Targeting Epigenetic Modifications in Cancer Therapy, Erasing the Roadmap to cancer.Pdf. Nat. Med. 25, 403–418. 30842676 10.1038/s41591-019-0376-8

[B60] Horn H. Ziepert M. Becher C. Barth T. F. Bernd H. W. Feller A. C. (2013). MYC Status in Concert with BCL2 and BCL6 Expression Predicts Outcome in Diffuse Large B-Cell Lymphoma. Blood 121 (12), 2253–2263. 10.1182/blood-2012-06-435842 23335369

[B61] Houzelstein D. Bullock S. L. Lynch D. E. Grigorieva E. F. Wilson V. A. Beddington R. S. (2002). Growth and Early Postimplantation Defects in Mice Deficient for the Bromodomain-Containing Protein Brd4. Mol. Cell Biol 22 (11), 3794–3802. 10.1128/mcb.22.11.3794-3802.2002 11997514 PMC133820

[B62] Howe L. R. Subbaramaiah K. Hudis C. A. Dannenberg A. J. (2013). Molecular Pathways: Adipose Inflammation as a Mediator of Obesity-Associated Cancer. Clin. Cancer Res. 19 (22), 6074–6083. 10.1158/1078-0432.CCR-12-2603 23958744 PMC3891839

[B63] Huang B. Yang X. D. Zhou M. M. Ozato K. Chen L. F. (2009). Brd4 Coactivates Transcriptional Activation of NF-kappaB via Specific Binding to Acetylated RelA. Mol. Cell Biol 29 (5), 1375–1387. 10.1128/MCB.01365-08 19103749 PMC2643823

[B64] Hussong M. Kaehler C. Kerick M. Grimm C. Franz A. Timmermann B. (2017). The Bromodomain Protein BRD4 Regulates Splicing during Heat Shock. Nucleic Acids Res. 45 (1), 382–394. 10.1093/nar/gkw729 27536004 PMC5224492

[B65] Jain A. K. Barton M. C. (2009). Regulation of P53: TRIM24 Enters the RING. Cell Cycle 8 (22), 3668–3674. 10.4161/cc.8.22.9979 19844164

[B66] Jang M. K. Mochizuki K. Zhou M. Jeong H. S. Brady J. N. Ozato K. (2005). The Bromodomain Protein Brd4 Is a Positive Regulatory Component of P-TEFb and Stimulates RNA Polymerase II-dependent Transcription. Mol. Cell 19 (4), 523–534. 10.1016/j.molcel.2005.06.027 16109376

[B67] Jeffers V. Yang C. Huang S. Sullivan W. J. (2017). Bromodomains in Protozoan Parasites: Evolution, Function, and Opportunities for Drug Development. Microbiol. Mol. Biol. Rev. 81 (1). 10.1128/MMBR.00047-16 PMC5312238 28077462

[B68] Kaikkonen M. U. Spann N. J. Heinz S. Romanoski C. E. Allison K. A. Stender J. D. (2013). Remodeling of the Enhancer Landscape during Macrophage Activation Is Coupled to Enhancer Transcription. Mol. Cell 51 (3), 310–325. 10.1016/j.molcel.2013.07.010 23932714 PMC3779836

[B69] Kanno T. Kanno Y. LeRoy G. Campos E. Sun H. W. Brooks S. R. (2014). BRD4 Assists Elongation of Both Coding and Enhancer RNAs by Interacting with Acetylated Histones. Nat. Struct. Mol. Biol. 21 (12), 1047–1057. 10.1038/nsmb.2912 25383670 PMC4720983

[B70] Kanno T. Kanno Y. Siegel R. M. Jang M. K. Lenardo M. J. Ozato K. (2004). Selective Recognition of Acetylated Histones by Bromodomain Proteins Visualized in Living Cells. Mol. Cell 13 (1), 33–43. 10.1016/s1097-2765(03)00482-9 14731392

[B71] Keating S. T. El-Osta A. (2013). Epigenetic Changes in Diabetes. Clin. Genet. 84 (1), 1–10. 10.1111/cge.12121 23398084

[B72] Kharenko O. A. Gesner E. M. Patel R. G. Norek K. White A. Fontano E. (2016). RVX-297- a Novel BD2 Selective Inhibitor of BET Bromodomains. Biochem. Biophys. Res. Commun. 477 (1), 62–67. 10.1016/j.bbrc.2016.06.021 27282480

[B73] Kharenko O. A. Patel R. G. Brown S. D. Calosing C. White A. Lakshminarasimhan D. (2018). Design and Characterization of Novel Covalent Bromodomain and Extra-terminal Domain (BET) Inhibitors Targeting a Methionine. J. Med. Chem. 61 (18), 8202–8211. 10.1021/acs.jmedchem.8b00666 30165024

[B74] Koo S. J. Fernández-Montalván A. E. Badock V. Ott C. J. Holton S. J. von Ahsen O. (2016). ATAD2 Is an Epigenetic Reader of Newly Synthesized Histone marks during DNA Replication. Oncotarget 7 (43), 70323–70335. 10.18632/oncotarget.11855 27612420 PMC5342555

[B75] Korb E. Herre M. Zucker-Scharff I. Darnell R. B. Allis C. D. (2015). BET Protein Brd4 Activates Transcription in Neurons and BET Inhibitor Jq1 Blocks Memory in Mice. Nat. Neurosci. 18 (10), 1464–1473. 10.1038/nn.4095 26301327 PMC4752120

[B76] Krivtsov A. V. Armstrong S. A. (2007). MLL Translocations, Histone Modifications and Leukaemia Stem-Cell Development. Nat. Rev. Cancer 7 (11), 823–833. 10.1038/nrc2253 17957188

[B77] Lamonica J. M. Deng W. Kadauke S. Campbell A. E. Gamsjaeger R. Wang H. (2011). Bromodomain Protein Brd3 Associates with Acetylated GATA1 to Promote its Chromatin Occupancy at Erythroid Target Genes. Proc. Natl. Acad. Sci. U S A. 108 (22), E159–E168. 10.1073/pnas.1102140108 21536911 PMC3107332

[B78] LeRoy G. Rickards B. Flint S. J. (2008). The Double Bromodomain Proteins Brd2 and Brd3 Couple Histone Acetylation to Transcription. Mol. Cell 30 (1), 51–60. 10.1016/j.molcel.2008.01.018 18406326 PMC2387119

[B79] Li D. Roberts R. (2001). WD-repeat Proteins: Structure Characteristics, Biological Function, and Their Involvement in Human Diseases. Cell Mol. Life Sci 58 (14), 2085–2097. 10.1007/pl00000838 11814058 PMC11337334

[B80] Liang K. Smith E. R. Aoi Y. Stoltz K. L. Katagi H. Woodfin A. R. (2018). Targeting Processive Transcription Elongation via SEC Disruption for MYC-Induced Cancer Therapy. Cell 175 (3), 766–e17. e17. 10.1016/j.cell.2018.09.027 30340042 PMC6422358

[B81] Lim S. L. Damnernsawad A. Shyamsunder P. Chng W. J. Han B. C. Xu L. (2019). Proteolysis Targeting Chimeric Molecules as Therapy for Multiple Myeloma: Efficacy, Biomarker and Drug Combinations. Haematologica 104 (6), 1209–1220. 10.3324/haematol.2018.201483 30606790 PMC6545861

[B82] Lin C. Y. Lovén J. Rahl P. B. Paranal R. M. Burge C. B. Bradner J. E. (2012). Transcriptional Amplification in Tumor Cells with Elevated C-Myc. Cell 151 (1), 56–67. 10.1016/j.cell.2012.08.026 23021215 PMC3462372

[B83] Liu D. Matzuk M. M. Sung W. K. Guo Q. Wang P. Wolgemuth D. J. (1998). Cyclin A1 Is Required for Meiosis in the Male Mouse. Nat. Genet. 20 (4), 377–380. 10.1038/3855 9843212

[B84] Liu W. Ma Q. Wong K. Li W. Ohgi K. Zhang J. (2013). Brd4 and JMJD6-Associated Anti-pause Enhancers in Regulation of Transcriptional Pause Release. Cell 155 (7), 1581–1595. 10.1016/j.cell.2013.10.056 24360279 PMC3886918

[B85] Lloyd J. T. Glass K. C. (2018). Biological Function and Histone Recognition of Family IV Bromodomain-Containing Proteins. J. Cell Physiol. 233 (3), 1877–1886. 10.1002/jcp.26010 28500727 PMC5683942

[B86] Lovén J. Hoke H. A. Lin C. Y. Lau A. Orlando D. A. Vakoc C. R. (2013). Selective Inhibition of Tumor Oncogenes by Disruption of Super-enhancers. Cell 153 (2), 320–334. 10.1016/j.cell.2013.03.036 23582323 PMC3760967

[B87] Mashtalir N. D'Avino A. R. Michel B. C. Luo J. Pan J. Otto J. E. (2018). Modular Organization and Assembly of SWI/SNF Family Chromatin Remodeling Complexes. Cell 175 (5), 1272–e20. e20. 10.1016/j.cell.2018.09.032 30343899 PMC6791824

[B88] McConkey D. J. Lee S. Choi W. Tran M. Majewski T. Lee S. (2010). Molecular Genetics of Bladder Cancer: Emerging Mechanisms of Tumor Initiation and Progression. Urol. Oncol. 28 (4), 429–440. 10.1016/j.urolonc.2010.04.008 20610280 PMC2901550

[B89] Mertz J. A. Conery A. R. Bryant B. M. Sandy P. Balasubramanian S. Mele D. A. (2011). Targeting MYC Dependence in Cancer by Inhibiting BET Bromodomains. Proc. Natl. Acad. Sci. U S A. 108 (40), 16669–16674. 10.1073/pnas.1108190108 21949397 PMC3189078

[B90] Mochizuki K. Nishiyama A. Jang M. K. Dey A. Ghosh A. Tamura T. (2008). The Bromodomain Protein Brd4 Stimulates G1 Gene Transcription and Promotes Progression to S Phase. J. Biol. Chem. 283 (14), 9040–9048. 10.1074/jbc.M707603200 18223296 PMC2431025

[B91] Monden T. Kishi M. Hosoya T. Satoh T. Wondisford F. E. Hollenberg A. N. (1999). p120 Acts as a Specific Coactivator for 9-Cis-Retinoic Acid Receptor (RXR) on Peroxisome Proliferator-Activated Receptor-Gamma/RXR Heterodimers. Mol. Endocrinol. 13 (10), 1695–1703. 10.1210/mend.13.10.0353 10517671

[B92] Morgado-Pascual J. L. Marchant V. Rodrigues-Diez R. Dolade N. Suarez-Alvarez B. Kerr B. (2018). Epigenetic Modification Mechanisms Involved in Inflammation and Fibrosis in Renal Pathology. Mediators Inflamm. 2018, 2931049. 10.1155/2018/2931049 30647531 PMC6311799

[B93] Morinière J. Rousseaux S. Steuerwald U. Soler-López M. Curtet S. Vitte A. L. (2009). Cooperative Binding of Two Acetylation marks on a Histone Tail by a Single Bromodomain. Nature 461 (7264), 664–668. 10.1038/nature08397 19794495

[B94] Mujtaba S. Zeng L. Zhou M. M. (2007). Structure and Acetyl-Lysine Recognition of the Bromodomain. Oncogene 26 (37), 5521–5527. 10.1038/sj.onc.1210618 17694091

[B95] Nickerson H. D. Joshi A. Wolgemuth D. J. (2007). Cyclin A1-Deficient Mice Lack Histone H3 Serine 10 Phosphorylation and Exhibit Altered aurora B Dynamics in Late Prophase of Male Meiosis. Dev. Biol. 306 (2), 725–735. 10.1016/j.ydbio.2007.04.009 17498682 PMC2701158

[B96] Nicodeme E. Jeffrey K. L. Schaefer U. Beinke S. Dewell S. Chung C. W. (2010). Suppression of Inflammation by a Synthetic Histone Mimic. Nature 468 (7327), 1119–1123. 10.1038/nature09589 21068722 PMC5415086

[B97] Okada Y. Feng Q. Lin Y. Jiang Q. Li Y. Coffield V. M. (2005). hDOT1L Links Histone Methylation to Leukemogenesis. Cell 121 (2), 167–178. 10.1016/j.cell.2005.02.020 15851025

[B98] Ortega E. Rengachari S. Ibrahim Z. Hoghoughi N. Gaucher J. Holehouse A. S. (2018). Transcription Factor Dimerization Activates the P300 Acetyltransferase. Nature 562 (7728), 538–544. 10.1038/s41586-018-0621-1 30323286 PMC6914384

[B99] Ott C. J. Kopp N. Bird L. Paranal R. M. Qi J. Bowman T. (2012). BET Bromodomain Inhibition Targets Both C-Myc and IL7R in High-Risk Acute Lymphoblastic Leukemia. Blood 120 (14), 2843–2852. 10.1182/blood-2012-02-413021 22904298 PMC3466965

[B100] Padmanabhan B. Mathur S. Manjula R. Tripathi S. (2016). Bromodomain and Extra-terminal (BET) Family Proteins: New Therapeutic Targets in Major Diseases. J. Biosci. 41 (2), 295–311. 10.1007/s12038-016-9600-6 27240990

[B101] Pan L. Zhu B. Hao W. Zeng X. Vlahopoulos S. A. Hazra T. K. (2016). Oxidized Guanine Base Lesions Function in 8-Oxoguanine DNA Glycosylase-1-Mediated Epigenetic Regulation of Nuclear Factor κB-driven Gene Expression. J. Biol. Chem. 291 (49), 25553–25566. 10.1074/jbc.M116.751453 27756845 PMC5207254

[B102] Patel M. C. Debrosse M. Smith M. Dey A. Huynh W. Sarai N. (2013). BRD4 Coordinates Recruitment of Pause Release Factor P-TEFb and the Pausing Complex NELF/DSIF to Regulate Transcription Elongation of Interferon-Stimulated Genes. Mol. Cell Biol. 33 (12), 2497–2507. 10.1128/MCB.01180-12 23589332 PMC3700095

[B103] Pérez-Salvia M. Esteller M. (2017). Bromodomain Inhibitors and Cancer Therapy: From Structures to Applications. Epigenetics 12 (5), 323–339. 27911230 10.1080/15592294.2016.1265710 PMC5453193

[B104] Pestell T. G. Jiao X. Kumar M. Peck A. R. Prisco M. Deng S. (2017). Stromal Cyclin D1 Promotes Heterotypic Immune Signaling and Breast Cancer Growth. Oncotarget 8 (47), 81754–81775. 10.18632/oncotarget.19953 29137220 PMC5669846

[B105] Picaud S. Wells C. Felletar I. Brotherton D. Martin S. Savitsky P. (2013). RVX-208, an Inhibitor of BET Transcriptional Regulators with Selectivity for the Second Bromodomain. Proc. Natl. Acad. Sci. U S A. 110 (49), 19754–19759. 10.1073/pnas.1310658110 24248379 PMC3856850

[B106] Rahman S. Sowa M. E. Ottinger M. Smith J. A. Shi Y. Harper J. W. (2011). The Brd4 Extraterminal Domain Confers Transcription Activation Independent of pTEFb by Recruiting Multiple Proteins, Including NSD3. Mol. Cell Biol. 31 (13), 2641–2652. 10.1128/MCB.01341-10 21555454 PMC3133372

[B107] Ray K. K. Nicholls S. J. Buhr K. A. Ginsberg H. N. Johansson J. O. Kalantar-Zadeh K. (2020). Effect of Apabetalone Added to Standard Therapy on Major Adverse Cardiovascular Events in Patients with Recent Acute Coronary Syndrome and Type 2 Diabetes: A Randomized Clinical Trial. Jama 323 (16), 1565–1573. 10.1001/jama.2020.3308 32219359 PMC7101505

[B108] Ray L. B. (2010). Metabolic Regulation through Acetylation. Science 3 (110), ec59. 10.1126/scisignal.3110ec59

[B109] Reyes-Garau D. Ribeiro M. L. Roué G. (2019). Pharmacological Targeting of BET Bromodomain Proteins in Acute Myeloid Leukemia and Malignant Lymphomas: From Molecular Characterization to Clinical Applications. Cancers (Basel) 11 (10), 1. 10.3390/cancers11101483 PMC6826405 31581671

[B110] Rhyasen G. W. Hattersley M. M. Yao Y. Dulak A. Wang W. Petteruti P. (2016). AZD5153: A Novel Bivalent BET Bromodomain Inhibitor Highly Active against Hematologic Malignancies. Mol. Cancer Ther. 15 (11), 2563–2574. 10.1158/1535-7163.MCT-16-0141 27573426

[B111] Savitsky P. Krojer T. Fujisawa T. Lambert J. P. Picaud S. Wang C. Y. (2016). Multivalent Histone and DNA Engagement by a PHD/BRD/PWWP Triple Reader Cassette Recruits ZMYND8 to K14ac-Rich Chromatin. Cell Rep. 17 (10), 2724–2737. 10.1016/j.celrep.2016.11.014 27926874 PMC5177622

[B112] Seeler J. S. Marchio A. Losson R. Desterro J. M. Hay R. T. Chambon P. (2001). Common Properties of Nuclear Body Protein SP100 and TIF1alpha Chromatin Factor: Role of SUMO Modification. Mol. Cell Biol. 21 (10), 3314–3324. 10.1128/MCB.21.10.3314-3324.2001 11313457 PMC100253

[B113] Shafran J. S. Andrieu G. P. Györffy B. Denis G. V. (2019). BRD4 Regulates Metastatic Potential of Castration-Resistant Prostate Cancer through AHNAK. Mol. Cancer Res. 17 (8), 1627–1638. 10.1158/1541-7786.MCR-18-1279 31110158 PMC6677600

[B114] Shang E. Nickerson H. D. Wen D. Wang X. Wolgemuth D. J. (2007). The First Bromodomain of Brdt, a Testis-specific Member of the BET Sub-family of Double-Bromodomain-Containing Proteins, Is Essential for Male Germ Cell Differentiation. Development 134 (19), 3507–3515. 10.1242/dev.004481 17728347

[B115] Shang E. Salazar G. Crowley T. E. Wang X. Lopez R. A. Wang X. (2004). Identification of Unique, Differentiation Stage-specific Patterns of Expression of the Bromodomain-Containing Genes Brd2, Brd3, Brd4, and Brdt in the Mouse Testis. Gene Expr. Patterns 4 (5), 513–519. 10.1016/j.modgep.2004.03.002 15261828

[B116] Shi J. Wang Y. Zeng L. Wu Y. Deng J. Zhang Q. (2014). Disrupting the Interaction of BRD4 with Diacetylated Twist Suppresses Tumorigenesis in Basal-like Breast Cancer. Cancer Cell 25 (2), 210–225. 10.1016/j.ccr.2014.01.028 24525235 PMC4004960

[B117] Shi J. Whyte W. A. Zepeda-Mendoza C. J. Milazzo J. P. Shen C. Roe J. S. (2013). Role of SWI/SNF in Acute Leukemia Maintenance and Enhancer-Mediated Myc Regulation. Genes Dev. 27 (24), 2648–2662. 10.1101/gad.232710.113 24285714 PMC3877755

[B118] Stanlie A. Yousif A. S. Akiyama H. Honjo T. Begum N. A. (2014). Chromatin Reader Brd4 Functions in Ig Class Switching as a Repair Complex Adaptor of Nonhomologous End-Joining. Mol. Cell 55 (1), 97–110. 10.1016/j.molcel.2014.05.018 24954901

[B119] Stonestrom A. J. Hsu S. C. Jahn K. S. Huang P. Keller C. A. Giardine B. M. (2015). Functions of BET Proteins in Erythroid Gene Expression. Blood 125 (18), 2825–2834. 10.1182/blood-2014-10-607309 25696920 PMC4424630

[B120] Stonestrom A. J. Hsu S. C. Werner M. T. Blobel G. A. (2016). Erythropoiesis Provides a BRD's Eye View of BET Protein Function. Drug Discov. Today Technol. 19, 23–28. 10.1016/j.ddtec.2016.05.004 27769353 PMC5116323

[B121] Suarez-Alvarez B. Rodriguez R. M. Ruiz-Ortega M. Lopez-Larrea C. (2017). BET Proteins: An Approach to Future Therapies in Transplantation. Am. J. Transpl. 17 (9), 2254–2262. 10.1111/ajt.14221 28173625

[B122] Subramanian A. Tamayo P. Mootha V. K. Mukherjee S. Ebert B. L. Gillette M. A. (2005). Gene Set Enrichment Analysis: a Knowledge-Based Approach for Interpreting Genome-wide Expression Profiles. Proc. Natl. Acad. Sci. U S A. 102 (43), 15545–15550. 10.1073/pnas.0506580102 16199517 PMC1239896

[B123] Sun R. Wu Y. Hou W. Sun Z. Wang Y. Wei H. (2017). Bromodomain-containing Protein 2 Induces Insulin Resistance via the mTOR/Akt Signaling Pathway and an Inflammatory Response in Adipose Tissue. Cell Signal 30, 92–103. 10.1016/j.cellsig.2016.11.011 27865874

[B124] Sun Y. Huang J. Song K. (2015). BET Protein Inhibition Mitigates Acute Myocardial Infarction Damage in Rats via the TLR4/TRAF6/NF-Κb Pathway. Exp. Ther. Med. 10 (6), 2319–2324. 10.3892/etm.2015.2789 26668635 PMC4665390

[B125] Tamkun J. W. Deuring R. Scott M. P. Kissinger M. Pattatucci A. M. Kaufman T. C. (1992). Brahma: a Regulator of Drosophila Homeotic Genes Structurally Related to the Yeast Transcriptional Activator SNF2/SWI2. Cell 68 (3), 561–572. 10.1016/0092-8674(92)90191-e 1346755

[B126] Tasdemir N. Banito A. Roe J. S. Alonso-Curbelo D. Camiolo M. Tschaharganeh D. F. (2016). BRD4 Connects Enhancer Remodeling to Senescence Immune Surveillance. Cancer Discov. 6 (6), 612–629. 10.1158/2159-8290.CD-16-0217 27099234 PMC4893996

[B127] Thirman M. J. Gill H. J. Burnett R. C. Mbangkollo D. McCabe N. R. Kobayashi H. (1993). Rearrangement of the MLL Gene in Acute Lymphoblastic and Acute Myeloid Leukemias with 11q23 Chromosomal Translocations. N. Engl. J. Med. 329 (13), 909–914. 10.1056/NEJM199309233291302 8361504

[B128] Ullah M. Pelletier N. Xiao L. Zhao S. P. Wang K. Degerny C. (2008). Molecular Architecture of Quartet MOZ/MORF Histone Acetyltransferase Complexes. Mol. Cell Biol. 28 (22), 6828–6843. 10.1128/MCB.01297-08 18794358 PMC2573306

[B129] Urbanucci A. Barfeld S. J. Kytölä V. Itkonen H. M. Coleman I. M. Vodák D. (2017). Androgen Receptor Deregulation Drives Bromodomain-Mediated Chromatin Alterations in Prostate Cancer. Cell Rep. 19 (10), 2045–2059. 10.1016/j.celrep.2017.05.049 28591577 PMC5675034

[B130] Urbanucci A. Mills I. G. (2018). Bromodomain-containing Proteins in Prostate Cancer. Mol. Cell Endocrinol. 462 (Pt), 31–40. 10.1016/j.mce.2017.06.007 28624514

[B131] Velíšek L. Shang E. Velíšková J. Chachua T. Macchiarulo S. Maglakelidze G. (2011). GABAergic Neuron Deficit as an Idiopathic Generalized Epilepsy Mechanism: the Role of BRD2 Haploinsufficiency in Juvenile Myoclonic Epilepsy. PLoS One 6 (8), e23656. 10.1371/journal.pone.0023656 21887291 PMC3161054

[B132] Wada T. Takagi T. Yamaguchi Y. Watanabe D. Handa H. (1998). Evidence that P-TEFb Alleviates the Negative Effect of DSIF on RNA Polymerase II-dependent Transcription *In Vitro* . Embo j 17 (24), 7395–7403. 10.1093/emboj/17.24.7395 9857195 PMC1171084

[B133] Wang F. Deeney J. T. Denis G. V. (2013). Brd2 Gene Disruption Causes “metabolically Healthy” Obesity: Epigenetic and Chromatin-Based Mechanisms that Uncouple Obesity from Type 2 Diabetes. Vitam Horm. 91, 49–75. 10.1016/B978-0-12-407766-9.00003-1 23374712 PMC3934552

[B134] Wang F. Liu H. Blanton W. P. Belkina A. Lebrasseur N. K. Denis G. V. (2009). Brd2 Disruption in Mice Causes Severe Obesity without Type 2 Diabetes. Biochem. J. 425 (1), 71–83. 10.1042/BJ20090928 19883376 PMC2819394

[B135] Wang L. Wolgemuth D. J. (2016). BET Protein BRDT Complexes with HDAC1, PRMT5, and TRIM28 and Functions in Transcriptional Repression during Spermatogenesis. J. Cell Biochem 117 (6), 1429–1438. 10.1002/jcb.25433 26565999 PMC4916496

[B136] Wang Q. Sun Y. Li T. Liu L. Zhao Y. Li L. (2019). Function of BRD4 in the Pathogenesis of High Glucose-induced C-ardiac H-ypertrophy. Mol. Med. Rep. 19 (1), 499–507. 10.3892/mmr.2018.9681 30483785 PMC6297744

[B137] White M. E. Fenger J. M. Carson W. E. 3rd (2019). Emerging Roles of and Therapeutic Strategies Targeting BRD4 in Cancer. Cell Immunol 337, 48–53. 10.1016/j.cellimm.2019.02.001 30832981 PMC6572734

[B138] Whyte W. A. Orlando D. A. Hnisz D. Abraham B. J. Lin C. Y. Kagey M. H. (2013). Master Transcription Factors and Mediator Establish Super-enhancers at Key Cell Identity Genes. Cell 153 (2), 307–319. 10.1016/j.cell.2013.03.035 23582322 PMC3653129

[B139] Winter G. E. Mayer A. Buckley D. L. Erb M. A. Roderick J. E. Vittori S. (2017). BET Bromodomain Proteins Function as Master Transcription Elongation Factors Independent of CDK9 Recruitment. Mol. Cell 67 (1), 5–e19. e19. 10.1016/j.molcel.2017.06.004 28673542 PMC5663500

[B140] Wu S. Y. Chiang C. M. (2007). The Double Bromodomain-Containing Chromatin Adaptor Brd4 and Transcriptional Regulation. J. Biol. Chem. 282 (18), 13141–13145. 10.1074/jbc.R700001200 17329240

[B141] Wu S. Y. Lee A. Y. Hou S. Y. Kemper J. K. Erdjument-Bromage H. Tempst P. (2006). Brd4 Links Chromatin Targeting to HPV Transcriptional Silencing. Genes Dev. 20 (17), 2383–2396. 10.1101/gad.1448206 16921027 PMC1560413

[B142] Wu S. Y. Lee A. Y. Lai H. T. Zhang H. Chiang C. M. (2013). Phospho Switch Triggers Brd4 Chromatin Binding and Activator Recruitment for Gene-specific Targeting. Mol. Cell 49 (5), 843–857. 10.1016/j.molcel.2012.12.006 23317504 PMC3595396

[B143] Wu S. Y. Nin D. S. Lee A. Y. Simanski S. Kodadek T. Chiang C. M. (2016). BRD4 Phosphorylation Regulates HPV E2-Mediated Viral Transcription, Origin Replication, and Cellular MMP-9 Expression. Cell Rep. 16 (6), 1733–1748. 10.1016/j.celrep.2016.07.001 27477287 PMC4981545

[B144] Xiao A. Li H. Shechter D. Ahn S. H. Fabrizio L. A. Erdjument-Bromage H. (2009). WSTF Regulates the H2A.X DNA Damage Response via a Novel Tyrosine Kinase Activity. Nature 457 (7225), 57–62. 10.1038/nature07668 19092802 PMC2854499

[B145] Xue J. Chen Y. Wu Y. Wang Z. Zhou A. Zhang S. (2015). Tumour Suppressor TRIM33 Targets Nuclear β-catenin Degradation. Nat. Commun. 6, 6156. 10.1038/ncomms7156 25639486 PMC4315364

[B146] Yamada T. Yamaguchi Y. Inukai N. Okamoto S. Mura T. Handa H. (2006). P-TEFb-mediated Phosphorylation of hSpt5 C-Terminal Repeats Is Critical for Processive Transcription Elongation. Mol. Cell 21 (2), 227–237. 10.1016/j.molcel.2005.11.024 16427012

[B147] Yang J. Chen S. Yang Y. Ma X. Shao B. Yang S. (2020). Jumonji Domain-Containing Protein 6 Protein and its Role in Cancer. Cell Prolif. 53 (2), e12747. 10.1111/cpr.12747 31961032 PMC7046477

[B148] Yang Z. He N. Zhou Q. (2008). Brd4 Recruits P-TEFb to Chromosomes at Late Mitosis to Promote G1 Gene Expression and Cell Cycle Progression. Mol. Cell Biol. 28 (3), 967–976. 10.1128/MCB.01020-07 18039861 PMC2223388

[B149] Yang Z. Yik J. H. Chen R. He N. Jang M. K. Ozato K. (2005). Recruitment of P-TEFb for Stimulation of Transcriptional Elongation by the Bromodomain Protein Brd4. Mol. Cell 19 (4), 535–545. 10.1016/j.molcel.2005.06.029 16109377

[B150] You J. Croyle J. L. Nishimura A. Ozato K. Howley P. M. (2004). Interaction of the Bovine Papillomavirus E2 Protein with Brd4 Tethers the Viral DNA to Host Mitotic Chromosomes. Cell 117 (3), 349–360. 10.1016/s0092-8674(04)00402-7 15109495

[B151] Zaware N. Zhou M. M. (2017). Chemical Modulators for Epigenome Reader Domains as Emerging Epigenetic Therapies for Cancer and Inflammation. Curr. Opin. Chem. Biol. 39, 116–125. 10.1016/j.cbpa.2017.06.012 28689146 PMC5581715

[B152] Zeng L. Yap K. L. Ivanov A. V. Wang X. Mujtaba S. Plotnikova O. (2008). Structural Insights into Human KAP1 PHD finger-bromodomain and its Role in Gene Silencing. Nat. Struct. Mol. Biol. 15 (6), 626–633. 10.1038/nsmb.1416 18488044 PMC3331790

[B153] Zeng L. Zhou M. M. (2002). Bromodomain: an Acetyl-Lysine Binding Domain. FEBS Lett. 513 (1), 124–128. 10.1016/s0014-5793(01)03309-9 11911891

[B154] Zhang A. Zheng C. Hou M. Lindvall C. Li K. J. Erlandsson F. (2003). Deletion of the telomerase reverse transcriptase gene and haploinsufficiency of telomere maintenance in Cri du chat syndrome. Am. J. Hum. Genet. 72 (4), 940–948. 10.1086/374565 12629597 PMC1180356

[B155] Zhang J. Dulak A. M. Hattersley M. M. Willis B. S. Nikkilä J. Wang A. (2018). BRD4 Facilitates Replication Stress-Induced DNA Damage Response. Oncogene 37 (28), 3763–3777. 10.1038/s41388-018-0194-3 29636547 PMC6101970

[B156] Zhang M. Y. Liu S. L. Huang W. L. Tang D. B. Zheng W. W. Zhou N. (2020). Bromodomains and Extra-terminal (BET) Inhibitor JQ1 Suppresses Proliferation of Acute Lymphocytic Leukemia by Inhibiting C-Myc-Mediated Glycolysis. Med. Sci. Monit. 26, e923411. 10.12659/MSM.923411 32266878 PMC7165247

[B157] Zhang Q. Zeng L. Shen C. Ju Y. Konuma T. Zhao C. (2016). Structural Mechanism of Transcriptional Regulator NSD3 Recognition by the ET Domain of BRD4. Structure 24 (7), 1201–1208. 10.1016/j.str.2016.04.019 27291650 PMC4938737

[B158] Zhao J. Q. Glasspool R. M. Hoare S. F. Bilsland A. Szatmari I. Keith W. N. (2000). Activation of Telomerase Rna Gene Promoter Activity by NF-Y, Sp1, and the Retinoblastoma Protein and Repression by Sp3. Neoplasia 2 (6), 531–539. 10.1038/sj.neo.7900114 11228546 PMC1508088

[B159] Zhao R. Nakamura T. Fu Y. Lazar Z. Spector D. L. (2011). Gene Bookmarking Accelerates the Kinetics of post-mitotic Transcriptional Re-activation. Nat. Cell Biol. 13 (11), 1295–1304. 10.1038/ncb2341 21983563 PMC3210065

[B160] Zhou Q. Li T. Price D. H. (2012). RNA Polymerase II Elongation Control. Annu. Rev. Biochem. 81, 119–143. 10.1146/annurev-biochem-052610-095910 22404626 PMC4273853

[B161] Zhu W. Wu R. D. Lv Y. G. Liu Y. M. Huang H. Xu J. Q. (2020). BRD4 Blockage Alleviates Pathological Cardiac Hypertrophy through the Suppression of Fibrosis and Inflammation via Reducing ROS Generation. Biomed. Pharmacother. 121, 109368. 10.1016/j.biopha.2019.109368 31707348

[B162] Ziai J. French C. A. Zambrano E. (2010). NUT Gene Rearrangement in a Poorly-Differentiated Carcinoma of the Submandibular Gland. Head Neck Pathol. 4 (2), 163–168. 10.1007/s12105-010-0174-6 20352379 PMC2878624

[B163] Zuber J. Shi J. Wang E. Rappaport A. R. Herrmann H. Sison E. A. (2011). RNAi Screen Identifies Brd4 as a Therapeutic Target in Acute Myeloid Leukaemia. Nature 478 (7370), 524–528. 10.1038/nature10334 21814200 PMC3328300

